# Retuning the Premedical Compass in American Programs Worldwide: Scoping Review

**DOI:** 10.2196/85002

**Published:** 2026-04-09

**Authors:** Michelle E Chouairy, Laetitia I Bou Samra, Christelle I Zeidan, Ola El Zein, Bilal R Kaafarani

**Affiliations:** 1 Medical Research Volunteer Program (MRVP), American University of Beirut, Beirut, Lebanon; 2 Clinical Research Institute, Faculty of Medicine, American University of Beirut, Beirut, Lebanon; 3 Department of Chemistry, American University of Beirut, PO Box 11-0236, Riad El Solh, Beirut, 1107-2020, Lebanon, +961-3151451

**Keywords:** American premedical curriculum, premedical education, revisiting premedical requirements, medical school preparedness, competency-based premedical curriculum, Medical College Admission Test, MCAT revision

## Abstract

**Background:**

Premedical education provides the academic foundation for entry into medical school, yet requirements differ widely across institutions and have shifted substantially over the past 2 decades. Recent Medical College Admission Test reforms and growing calls for more comprehensive and interdisciplinary premedical training have triggered curricular changes fragmented throughout the literature. Such manifestations make it particularly difficult for institutions and students to interpret the direction, rationale, and effectiveness of these changes.

**Objective:**

This scoping review aims to map the literature in order to identify key reforms in premedical course requirements and classify these changes into different types: addition of courses, removal of courses, redesign of courses, and the proposition of a new curriculum. This review also evaluates the motives, rationales, and outcomes of these changes for the sake of building a unified foundation for institutions, stakeholders, and students aspiring to delve into medical education.

**Methods:**

We systematically searched 4 databases up to July 4, 2025. We included studies focusing on American undergraduate premedical curricula and reporting changes to course or curriculum requirements. Studies discussing non-American programs, medical or postmedical curricula, or requirements beyond courses were excluded. Two reviewers independently screened studies at the title, abstract, and full-text levels, with conflicts resolved by consensus among all team members. Data extraction was also performed in duplicates. The Mixed Methods Appraisal Tool (McGill University) was used to assess the risk of bias. Because of its heterogeneity, the data were synthesized narratively.

**Results:**

We included 70 studies, which ranged across mixed methods, quantitative, qualitative, and descriptive designs. Three recurring drivers of reform emerged: conforming to Medical College Admission Test revisions, educating and forming well-prepared aspiring physicians, and transitioning to competency-based education. From the 70 studies, 36 highlighted adding courses, 25 described revising existing courses, 5 discussed course deletions, and 10 proposed new curricula. Outcomes prevalently showed improved preparedness for medical school, positive student feedback, and enhanced academic performance, though some results were mixed.

**Conclusions:**

To our knowledge, there is no comprehensive review on how the premedical curriculum has evolved over time. Thus, this scoping review, encompassing all these changes, will be a unified framework that may help both institutions and students become aware of the premedical course requirements on a broader scale and determine which reforms are most effective. Since 2000, premedical education has undergone a shift from a narrow science checklist toward a more comprehensive outcomes-oriented preparation, whereby a balanced approach that merges scientific core courses with social sciences and humanities seems to be the most promising for preparing premedical students for medical school and contributing to their professional identity formation. Future work should investigate to what extent these changes to the premedical course requirements have been implemented.

## Introduction

### Rationale

Many students aspiring to become doctors choose to follow the American program whether at an institution in the United States or abroad. This American-style pathway is widely adopted internationally, yet what constitutes “adequate” premedical preparation is not standardized across institutions. This program is characterized by a structured premedical education track, an academic foundation that ensures students enter medical school prepared and ready. The latter includes the completion of an undergraduate degree alongside essential premedical course requirements in general biology, chemistry, physics, English, and other subjects necessary to prepare students for the Medical College Admission Test (MCAT), a standardized multiple-choice exam that assesses students’ readiness for admission to medical school.

Historically, premedical education was shaped by the 1910 Flexner Report, which highlighted the necessity of establishing a premedical curriculum centered on natural sciences like physics, biology, and chemistry [1]. At that time, these courses were considered tools to “weed out” less-prepared medical school applicants. However, recent critiques and contemporary reports, such as those from the Howard Hughes Medical Institute–Association of American Medical Colleges, have emerged and called for greater emphasis on liberal arts, humanities, and ethics in premedical education [2]. Over the years, several revisions to the MCAT have been attempted, with the most recent occurring in 2015 [3]. Pertinent to that is the shift in the framework from a rigid course “checklist” toward competencies and outcomes-oriented preparation.

Indeed, in 2015, the Association of American Medical Colleges revised the MCAT and included psychology and sociology critical analysis sections, shifting the premedical focus from natural sciences to broader critical thinking skills [4]. Importantly, these changes in the MCAT altered the narrative of scientific preparation by shedding light on biochemistry and research methods, whereas the previous exam focused on discrete knowledge within chemistry and biology [5]. The new version required students to combine these disciplines in order to solve complex problems that serve to mirror the interdisciplinary nature of modern medical curricula. Additionally, the substitution of “verbal reasoning” with critical analysis and reasoning skills further expanded the scope of premedical inquiry into the humanities and social sciences, while prioritizing the ability to synthesize diverse information over simple reading comprehension skills. Consequently, this pathway forced undergraduate institutions to move away from “premedical checklists” in light of curricula that instigate scientific inquiry and interdisciplinary reasoning [5]. Ultimately, this redesign explicitly broadened the framework for assessing readiness by integrating behavioral and social sciences and emphasizing reasoning skills. Nevertheless, these changes created a disconnect between what is tested on the MCAT, what is required for admission by different medical schools, and what is taught in undergraduate institutions. This misalignment may be amplified by the persistent heterogeneity in prerequisite structures across medical schools.

Changes in the MCAT have led institutions, educators, physicians, and even students to question the content, purpose, and structure of their undergraduate curricula that differs from one institution to another. The undergraduate curriculum of the American program must be fixed, and various measures and reforms are taking place in many universities around the world. Growing critiques are found in the literature, as rethinking premedical education might not be evolving in the right direction. The literature is filled with studies discussing how different institutions are reevaluating their curricula and changing their premedical requirements. However, the literature available is fragmented. In fact, competency-based education has increasingly influenced how readiness could be connotatively defined, with newer Association of American Medical Colleges frameworks emphasizing broader competencies, such as thinking or reasoning, interpersonal skills, and professional development alongside the scope of scientific foundations [4]. Ultimately, recent scholarships have also reevaluated the educational value of the humanities and broader interdisciplinary preparation for professional identity formation (PIF) in premedical students, preparing them for “thinking, acting, and feeling like physicians.” Indeed, PIF refers to the developmental process through which students internalize the values, ethical considerations, behaviors, and norms of the profession they aspire to practice [6].

### Objectives

Motivated by the need to understand the nature and impact of recent modifications to premedical course requirements across the literature, this scoping review may serve as a guide for educators, curriculum designers, medical schools, and students themselves to identify the best combination of courses needed to fulfill the academic requirements for medical education. The aim of this scoping review is to answer the following question: How have premedical course requirements in American undergraduate programs changed since the year 2000, and what are the motives and outcomes associated with these modifications?

Through an intricate screening process of the available literature, this scoping review provides an evidence-based, unbiased synthesis of the most effective changes to the premedical curriculum according to their outcomes and impact on students’ admission to medical school and future academic performance. This scoping review aims to classify the changes in the literature into 4 types: the addition of courses to fulfill a certain need [1]; the removal of courses that do not affect students’ preparation for medical school [2,7]; the redesign of courses to tailor them more to the premedical students’ needs [8]; and the proposition of a new curriculum that no longer includes traditional premedical course requirements, such as a new major designed specifically for premedical student preparation [2,9-11]. Each of these changes’ drivers, outcomes, and rationales will also be reviewed and examined to build a unified synthesis of the available literature. By consolidating this fragmented evidence base, our findings are directed to support decision-making for institutions seeking to modernize premedical requirements and for students navigating variable prerequisites across programs.

## Methods

### Protocol and Registration

This scoping review critically maps and synthesizes the literature on premedical requirements in American programs worldwide. We registered the protocol for this scoping review on the Open Science Framework, adhering to the PRISMA-ScR (Preferred Reporting Items for Systematic Reviews and Meta-Analyses Extension for Scoping Reviews) guidelines (Checklist 1) [12].

### Search Strategy and Information Sources

We individually searched the following 4 electronic databases on December 16, 2024, and then reran the search up until July 4, 2025, in accordance with the PRISMA-S (Preferred Reporting Items for Systematic Reviews and Meta-Analyses Literature Search Extension) guidelines (Checklist 2), without language limitations: MEDLINE (using Ovid), Embase (using Elsevier), ERIC, and Education Research Complete (using EBSCO) [13]. The search strategy included both controlled vocabularies (eg, Medical Subject Headings [MeSH] and Emtree) and keywords such as premedical requirements, premedical education, prerequisite courses for medical school, premedical students, school admission criteria, premedical acceptance, premedical curriculum, premedical courses, prerequisite, precondition, and applying for medical school. This review did not involve searching study registries, applying predefined search filters, or conducting a formal peer review of the search strategy. The search strategies were not adapted from other reviews; they were developed by the director of evidence synthesis (OZ) at the American University of Beirut (AUB). The full search strategies for MEDLINE, Embase, ERIC, and Education Research Complete are provided in Multimedia Appendices 1-4. As for gray literature, both backward and forward citation tracking of all relevant studies was performed. The Howard Hughes Medical Institute–Association of American Medical Colleges reports were purposefully searched, but no additional data were sought by contacting authors, experts, manufacturers, or others.

### Eligibility Criteria

**Figure 1.**
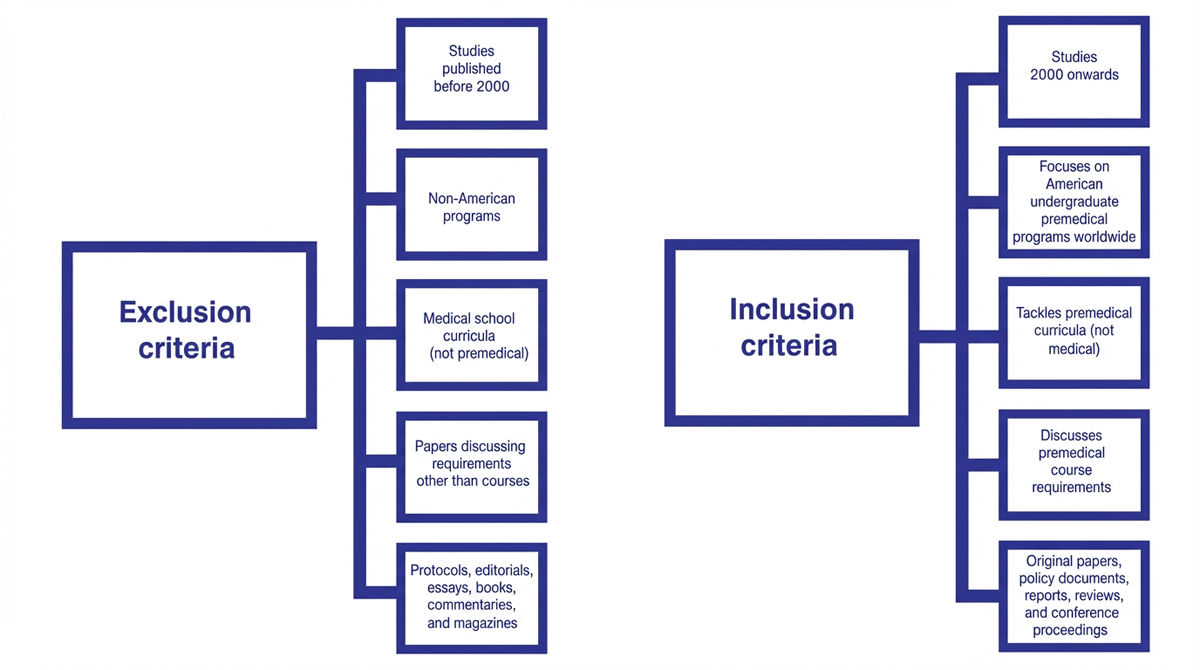
Exclusion and inclusion criteria.

The studies were selected based on a specific set of inclusion and exclusion criteria, as shown in Figure 1. Studies were included if they were: (1) published from 2000 onwards; (2) focused on American undergraduate premedical programs worldwide; (3) tackled premedical curricula (not medical); (4) discussed premedical course requirements; and (5) identified as original studies, policy documents, reports, reviews, or conference proceedings.

Studies were excluded if they were: (1) published before 2000; (2) tackled non-American programs; (3) discussed medical and not premedical curricula; (4) focused on requirements other than courses, such as extracurriculars, internships, and research; or (5) were identified as a protocol, editorial, erratum, essay, book, commentary, or magazine article.

### Selection of Sources of Evidence

All studies were imported into Covidence (Veritas Health Innovation Ltd), an online software platform designed to improve the efficiency and experience of creating and maintaining scoping reviews [14]. The platform was also used to detect and remove duplicates.

### Data Charting Process

Pairs of researchers (MEC, LIB, and CIZ) independently performed title, abstract, and full-text screening in duplicates, and after calibration exercises, reached a 95% agreement rate among reviewers. Disagreements among reviewers were resolved through discussions among all team members. Duplicate entries were removed manually and by Covidence before screening. The reasons for excluding studies at the full-text level were documented and shown in the PRISMA (Preferred Reporting Items for Systematic Reviews and Meta-Analyses) flowchart.

### Data Extraction Process

Pairs of researchers (MEC, LIB, and CIZ) conducted data extraction independently and in duplicates using a standardized table developed specifically for this review and piloted on a small subset of included studies. For each study, we abstracted the following variables: author, year, location, institution, type of change (add, remove, revise, or new curriculum), motives, outcomes, and themes of each study, which were compiled into a Microsoft Excel table. A unified and comprehensive table was synthesized after resolving any discrepancies in the extracted information through consensus (Multimedia Appendix 5).

### Critical Appraisal

The Mixed Methods Appraisal Tool (MMAT; McGill University) was used for quality and risk of bias assessment. This method allows for the appraisal and description of methodological quality across 3 domains: mixed, qualitative, and quantitative (partitioned into 3 subdomains: randomized controlled, nonrandomized, and descriptive) [15]. The risk of bias assessment was completed, and the findings were compiled in an Excel spreadsheet (Multimedia Appendix 6).

### Synthesis of Results

The data from the included studies were synthesized narratively. Studies were grouped according to 3 key themes related to the addition, removal, or modification of a course. Outcomes were categorized into 4 types: positive feedback about the course, positive impact and improvement for future career development or medical school, mixed outcomes, or new insights about the course.

### Ethical Considerations

Ethical approval was not required as this study was a scoping review.

## Results

### Selection of Sources of Evidence

**Figure 2.**
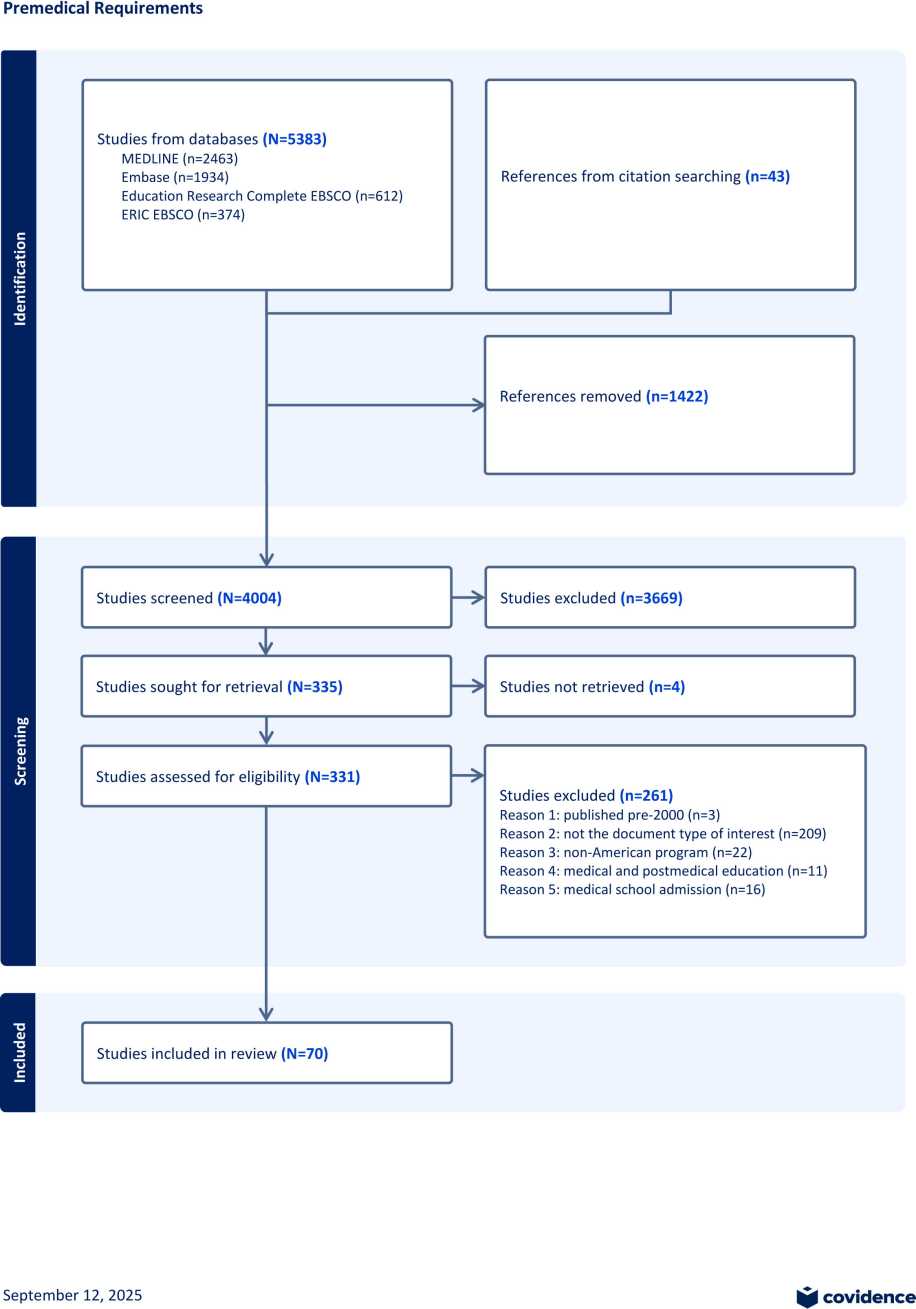
PRISMA (Preferred Reporting Items for Systematic Reviews and Meta-Analyses) flowchart.

The search strategy and citation tracking retrieved 4004 studies after duplicate removal. We conducted title and abstract screening, leaving 335 studies for full-text screening. Following full-text screening, 261 studies were excluded for not meeting the selection criteria defined by specific reasons. Three references were excluded under reason 1 (published pre-2000), 209 references were excluded under reason 2 (not the document type of interest), 22 under reason 3 (non-American programs), 11 under reason 4 (medical and postmedical education), and 16 under reason 5 (medical school admission). Four studies lacked accessible full texts and were subsequently excluded at this stage. Accordingly, 70 studies were included in data extraction, analysis, and quality assessment (Figure 2).

### Characteristics of Sources of Evidence

**Figure 3.**
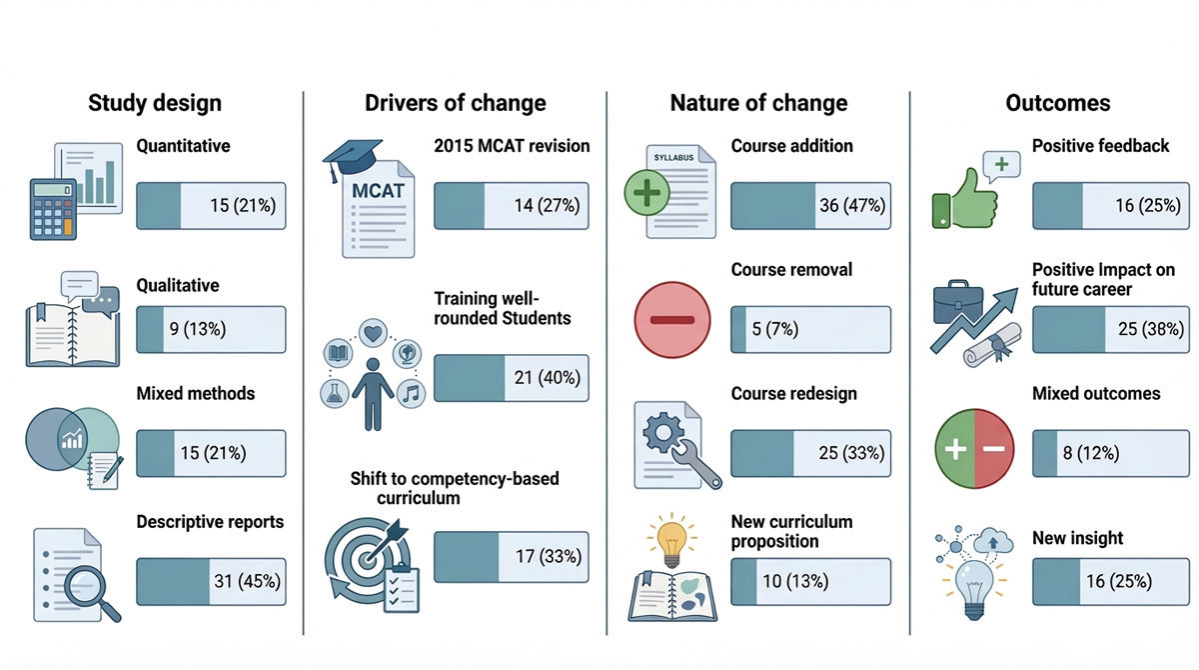
Summary characteristics of studies reviewed (N=70). Percentages are calculated based on the total number of studies included. Counts may not sum to 70 in all categories due to multiple categorizations or rounding. The figure was generated by FigureLabs. MCAT: Medical College Admission Test.

The included studies were published from 2000 onwards, predominantly in the United States, with occasional exceptions (1 in Australia [16] and 1 in Qatar [17]). Study types comprised 15 mixed methods studies, 15 quantitative studies, 9 qualitative studies, and 31 descriptive reports that were not eligible for MMAT risk of bias assessment. All institutions involved in these studies were universities following the American undergraduate premedical track (Figure 3).

### Critical Appraisal Within Sources of Evidence

The remaining 39 studies that qualified as quantitative, qualitative, or mixed methods studies were appraised in Excel using MMAT [15]. Following the risk of bias assessment, 3 studies that did not effectively explain the relevance of their outcomes to course or curriculum reforms were excluded [18-20]. Quality scores ranged from meeting none of the 5 criteria (0) to meeting all 5 criteria (5). The risk of bias assessment results are provided in Multimedia Appendix 6. Each study’s design, outcomes, changes to courses, and drivers of change are reported in Multimedia Appendix 5.

### Synthesis of Results

#### Main Drivers of Change

Across the 67 remaining studies, 3 recurring drivers of curriculum reform or course modification were identified. First, the demand for medical schools to accept well-rounded students (n=21, 40%; Figure 3) drove the integration of courses emphasizing empathy [21], patient-physician interactions [22], and general education, such as global health [23]. Second, a shift from course-based to competency-based education (n=17, 33%) encouraged institutions to implement interdisciplinary classes [24] and specialized courses focusing on technical [25] and interpersonal skills [22]. Finally, a significant driver was the 2015 MCAT revision (n=14, 27%; Figure 3), which introduced behavioral and social sciences as well as critical reasoning sections. This motivated institutional reforms, as every college sought to prepare students to succeed in the standardized exam indispensable for continuing a career in medicine. Universities responded by introducing courses in reflective writing [26], psychology [1,10,17,27,28], sociology [7,29,30], and anthropology [3,27].

#### Main Natures of Change

All the reforms resulting from these convergent institutional motives clustered into 4 categories, thus characterizing the nature of the changes made. Particularly, 36 (47%) studies (Figure 3) focused on adding new courses to the premedical curriculum, in which the most frequent additions were courses in anatomy [31-35], psychology [1,10,17,27,28], statistics [1,27], histology [31,33], and mathematics [2,36]. On the other hand, other studies introduced specialized topics such as neuroimmune pharmacology [37], point-of-care ultrasound [25], childhood obesity [38], microbiology [39], evolution [40-42], and antibiotic resistance [43]. Simultaneously, several studies also emphasized making humanities-based subjects such as global health [27], medical anthropology [3,27], and sociology [17,27-29] required for premedical students to better prepare them for medical school (Multimedia Appendix 5).

From the 67 studies, 25 (33%; Figure 3) addressed redesigning, revising, or replacing existing courses to align with the shift toward competency-based tracks. For example, physics [1,44-50], sociology [17,28,30], biochemistry [40,51-53], and biology [27,40,53,54] courses were most often targeted for redesign because their content was perceived as diverging from their primary purpose: ensuring student readiness for medical school admission. Some institutions proposed redesigning courses by integrating laboratory components (cadaver [55] and dissection anatomy and physiology labs [56,57]) or changing course duration (intensive general chemistry in 1 semester [58]; Multimedia Appendix 5).

Furthermore, 10 (13%) studies (Figure 3) proposed new curricula or competency-based sequences [2,9-11] to help students succeed by building interdisciplinary connections and interpersonal skills. These studies demonstrated that some students could thrive without a traditional science background, as seen in programs such as the Humanities and Medicine Program (HuMed) [59,60]. Particularly, some institutions also designed new majors in biology [61,62] and biochemistry [8] specifically tailored for premedical students and aspiring physicians. Essentially, these programs aimed to achieve a more flexible and comprehensive curriculum that would better fulfill students’ needs and cultivate diverse skills (Multimedia Appendix 5). This curriculum included courses tailored to meet premedical students’ needs while preparing them for medical school.

It is also critical to note that only 5 out of 67 (7%) studies (Figure 3) examined removing courses from the undergraduate premedical track. For instance, chemistry (organic [2,7] and general [58]) and calculus [7], traditionally viewed as “weed-out” courses by medical school admissions committees, were the main targets. These reports argued that these courses did not benefit students; instead, they harmed them by adding stress to an already competitive premedical journey (Multimedia Appendix 5).

**Figure 4.**
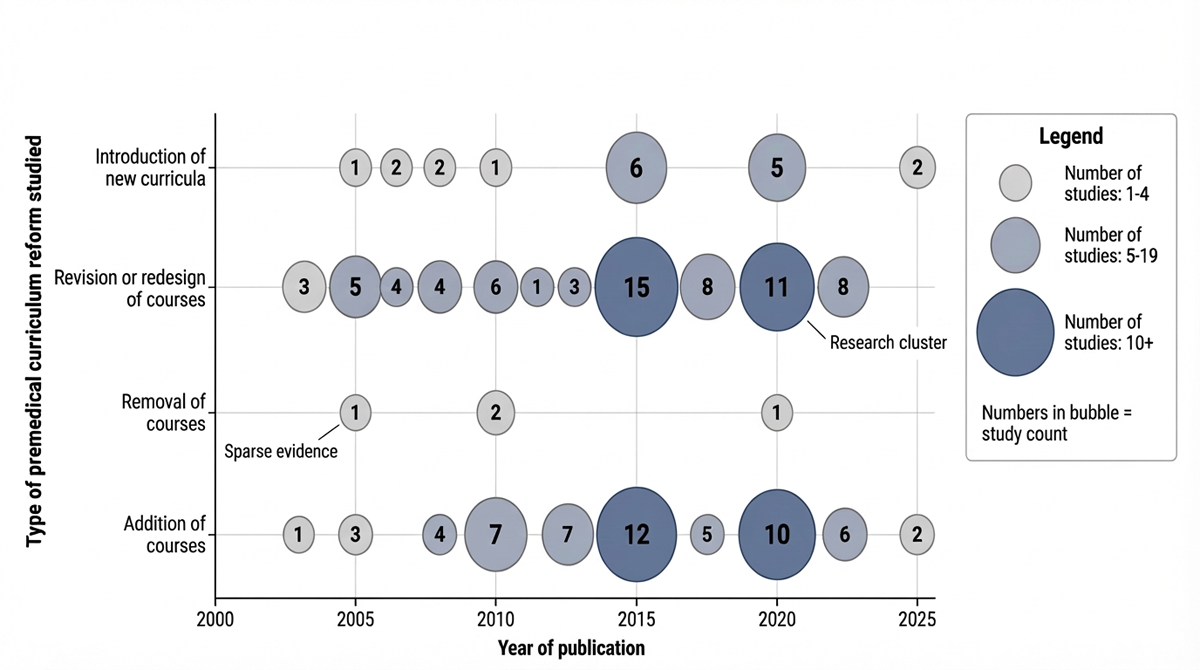
Evidence gap map of studies examining premedical curriculum reforms in American programs worldwide (2000‐2025). Each bubble represents the number of studies in a given year and reform category; bubble size and embedded numbers reflect the study count. The figure was generated by FigureLabs.

Moreover, an evidence gap map was generated to summarize the data collected from these references and to visualize the distribution of these studies clearly (Figure 4). The map showcases the pattern of changes to premedical requirements worldwide from 2000 onwards. It aids in identifying areas of strong evidence, such as research clusters of studies examining course redesign and additions, particularly after 2010. Additionally, it highlights domains like the removal of courses and the proposition of a new curriculum, where research remains limited and future investigation is needed to further support these findings. Finally, this map shows a temporal increase in evidence availability after 2010, with the highest concentration of studies occurring between 2015 and 2020 (Figure 4).

#### Main Outcomes

Indeed, a significant pattern of outcomes was retrieved across the 67 analyzed studies, in which a large number emphasized the positive impact of these changes on future career development and medical school preparedness (n=25, 38%; Figure 3) by comparing grade point average [22,32], rate of acceptance [59], step 1 scores [59], as well as future performance in medical school [31,32,60,63]. Additionally, the incorporation of anatomy courses into the premedical curriculum was proven to be a very good predictor of improvement at the start of the medical school journey (Multimedia Appendix 5) [31,32]. Consequently, well-designed premedical courses can significantly support career development and guide students toward a successful path.

Another apparent outcome encountered was essentially positive feedback regarding the course (n=16, 25%; Figure 3), especially in studies that underpinned the interdisciplinary approaches. In fact, students expressed that integrating different subjects, such as biology and mathematics, was more interactive [24]. Moreover, additional positive feedback was solicited from students who were taught anatomy and physiology through active, hands-on approaches (Multimedia Appendix 5) [55,56]. Additionally, students valued the integration of humanities into the undergraduate premedical years and had a positive attitude regarding this addition to the premedical curriculum [26]. In all the aforementioned cases, the changes served to increase student satisfaction and motivation.

To continue, several studies have proposed new insights into courses and curricula (n=16, 25%; Figure 3), specifically when measurable outcomes were limited. The perceived versus actual use of mathematics in medicine instigates a gap between what students believe about the use of mathematics and what practicing physicians actually report, thereby offering a completely new rationale for curricular reform [36]. Simultaneously, a study by Watkins et al [54] provided valuable lessons about aligning physics curricula with the practices of biomedical science (Multimedia Appendix 5).

Finally, it is important to note that some studies reported negative outcomes as well. Particularly, mixed findings (n=8, 12%; Figure 3) were observed, where benefits were achieved in certain contexts but not in others. For example, after taking undergraduate-level anatomy, undergraduate premedical students concluded that, while they valued anatomy courses, the reform did not lead to measurable, quantified improvements in future academic performance [34]. Essentially, while reforms may fuel engagement and self-perceived readiness, they do not always yield significant changes in traditional academic outcomes such as grades or standardized test scores, and ultimately, medical school preparedness and acceptance (Multimedia Appendix 5) [7,16,35,39,60,64,65].

## Discussion

### Summary of Evidence

This scoping review maps and synthesizes evidence from 70 studies published from 2000 onwards that discuss reforms to premedical course requirements in American programs worldwide. Our scoping review screens the literature to identify these reforms, classify the types of changes, and evaluate the motives, rationales, and outcomes of these changes for the sake of building a unified foundation for institutions, stakeholders, and students aspiring to delve into medical education. Our findings underscore the most common drivers of curricular change pertaining to the 2015 MCAT revision, the increasing importance of preparing well-trained aspiring physicians, as well as the shift toward competency-based education. Particularly, institutions opted to add new courses such as, but not limited to, anatomy [31-35], psychology [1,10,17,27,28], statistics [1,27], and humanities [17,27-29]; revise traditional ones to integrate clinical or laboratory components; and, in some cases, dilute some long-standing courses such as organic chemistry [2,7] and remove others like calculus [7]. Across these interventions, the prevailing outcome was a perceived or, at times, measurable improvement in student preparedness for medical school.

### Interpretations

In fact, the 2015 MCAT revision played a significant role in reshaping the plight of US premedical requirements. These revisions to the MCAT drew scholarly attention to premedical curriculum reforms during this period, explaining the concentration of studies occurring between 2015 and 2020 (Figure 4). The 2015 New MCAT retuned the requirements in terms of preparation for competencies in biochemistry, behavioral and social sciences, and critical analysis, thus nudging programs from legacy checklist models toward competency-oriented design. Indeed, recent reports framed the exam as both a signal of evolving expectations that institutions could use to justify curricular and advising changes [1,28,66]. The 2015 MCAT brought about a shift toward evidence-informed, competency-focused preparation, with the strongest momentum coming from local pilots reporting concrete learning advantages [47,52,67]. Nonetheless, although the MCAT has endured a broader competency profile, misalignment persists between what is tested and what is required. For example, sociology and psychology remain “recommended” rather than required courses at many institutions, leaving students to discover these expectations later through advising or even peers [29]. To illustrate, recent US commentaries also note that applicants can be “MCAT-ready but list-deficient” (or the reverse), depending on the target school, which is essentially an artifact of decentralized governance and historical precedent [1,66,68]. The downstream effect is avoidable inequity: students at campuses with strong prehealth advising or clear catalogs are anticipated to front-load MCAT-tested domains, while others may take the exam without formal exposure to key content [29]. Indeed, evidence from redesigned science sequences (eg, life-science physics and compact general chemistry) suggests that fit-for-purpose courses can close this gap while preserving rigor, but it is undeniable that adoption remains uneven [9,47,58,67].

Furthermore, while it is clear that a paradigm shift in premedical curricula is essential, many programs are reluctant to embrace these changes due to multiple obstacles. Some of the crucial challenges are clustered around legacy course loyalty, departmental identity, coordination costs, and ambiguity regarding equivalencies. Indeed, faculty and staff report concerns about reduced rigor in relation to challenging traditional requirements [59,66]. It is also important to note attempts to integrate premedical preparation with medical curricula, which have reported incentive misalignments, assessment mismatches, and governance friction across undergraduate and professional schools [31]. Finally, anchoring social or behavioral foundations in the early years (with clear MCAT linkages) is essential to bridge the gap between what students learn and how they are assessed by medical school admissions committees [29,35]. To embrace these changes, coordination between medical schools and undergraduate colleges is essential to ensure the implemented changes align with the competencies they look for in potential applicants.

### Comparison With Existing Literature

Our results are consistent with previous reports that have questioned the adequacy of traditional premedical curricula dominated by natural sciences [29,31,41,68]. On one hand, the pattern of reforms unraveled in this review mirrors broader shifts in medical education, shedding light on the integration of the humanities, ethics, and social sciences to cultivate empathy, cultural competence, and critical reasoning [26,38]. On the other hand, reforms were aligned with physics, chemistry, and biology within biomedical contexts, resonating with the literature and stressing the need for disciplinary authenticity—that is, aligning traditional foundational science knowledge with medical applications using interdisciplinary approaches [24,25,43,63]. Consequently, these trends together reflect a shift from content rigid coursework to competency-based curricula that integrate scientific knowledge and concrete applications. This substantiates the hypothesis that targeted course reform can foster not only cognitive readiness but also PIF at the undergraduate stage [21,50]. Hence, premedical students applying to medical school will have undergone a developmental process through which they internalize the values, roles, and responsibilities of the medical profession [6,21,50]. Collectively, these reforms suggest that integrating scientific coursework with humanistic competencies better prepares students for the MCAT exam, medical school admission, and professional practice. Competency-based education emphasizes that professional identity encompasses not only theoretical scientific knowledge but also attributes such as empathy, integrity, resilience, beneficence, and proper patient-centered care [6]. Consequently, retuning the premedical compass expands the concept of readiness beyond academic excellence aligning with the evolution of the MCAT toward assessing social, behavioral, and critical thinking domains.

### Strengths

This review has valuable strengths. It is essentially the first, to our knowledge, to comprehensively examine course-level reform in premedical education across 2 and a half decades. The inclusion of both American and international factions following the American program greatly enhances its representativeness. Furthermore, the large number of studies that were included, combined with the systematic methodology used to find these studies, provides a strong foundation for synthesizing fragmented literature.

### Limitations

However, limitations must be acknowledged; considerable heterogeneity in study designs, outcomes, and reporting limited quantitative synthesis. A significant portion of the studies was not eligible for MMAT critical appraisal; many relied on self-reported student feedback or descriptive accounts without rigorous statistical evaluation, thereby raising concerns about response and publication bias [21,25,26,29]. In addition, studies addressing the removal of courses are scarce over the entire 25-year period, and evidence appears to be very limited. Furthermore, research on the proposition of new curricula remains relatively limited compared to other types of changes (Figure 4). This indicates that further investigation is needed to fully determine which courses need to be removed and whether proposing new curricula is a good approach to best prepare premedical students for medical school.

### Implications

Regardless of these caveats, the findings hold tangible implications. For students, greater transparency in premedical requirements afforded by institutions to benefit students could reduce uncertainty and promote earlier academic planning [65,68]. For educators and institutions, greater transparency and better implementation of changes based on evidence could improve the quality of education provided to these premedical students [18,41,45]. At a policy level, medical schools and accrediting bodies should consider harmonizing admission requirements with both MCAT content and undergraduate course offerings to reduce misalignment and student burden [20,27,60]. Practically, longitudinal research points toward understanding the essentiality of the link between undergraduate course exposure to medical school and career outcomes [31]. Essentially, more evidence is needed to quantify to what extent the changes in these courses were implemented to provide students with a comprehensive foundation for medical school. That is why future research should prioritize prospective, longitudinal studies that measure both academic and nonacademic outcomes, including well-being and patient-related competencies [69-75].

### Conclusions

In conclusion, our review encompasses all the changes fragmented in the literature and accentuates that targeted reforms to premedical course requirements can enhance preparedness for medical school. However, it is critical to note that their impact is uneven and context dependent. The latter necessitates a rather balanced approach, integrating rigorous scientific foundations with the humanities and social sciences, which appears most promising for shaping the next generation of physicians prepared both academically and professionally. This review is innovative because it moves beyond isolated institutional descriptions and commentary by consolidating 70 sources into a single evidence map of premedical course–requirement reform since 2000 across American programs worldwide, explicitly linking reform types to their stated drivers and outcomes. Specifically, what this review brings to the field is a transferable and dynamic framework that helps interpret a fragmented literature and highlights persistent misalignment between the discrepancy in what is being tested, taught, and required—a pertinent issue with equity consequences when access to advising and coursework varies across institutions. In reality, these findings can inform curriculum committees and program leaders designing updated premedical tracks, support advising strategies that anticipate heterogeneous medical school requirements, and provide admissions stakeholders with a clearer understanding of how prerequisite policies interact with competency-focused readiness, thus helping to reduce avoidable gaps in preparation for students across diverse educational contexts. Therefore, essential future work aligning prerequisites to 2015 MCAT competencies, coupled with advising clarity and outcome reporting to understand the extent to which these course requirements have been implemented, would significantly offer a pathway to equity, efficiency, and preparedness without sacrificing scientific rigor.

## Supplementary material

10.2196/85002 Multimedia Appendix 1 The full MEDLINE search terms used in the review.

10.2196/85002 Multimedia Appendix 2 The full Embase search terms used in the review.

10.2196/85002 Multimedia Appendix 3 The full ERIC search terms used in the review.

10.2196/85002 Multimedia Appendix 4 The full Education Research Complete search terms used in the review.

10.2196/85002 Multimedia Appendix 5 Comprehensive data extraction table.

10.2196/85002 Multimedia Appendix 6 Mixed Methods Appraisal Tool (MMAT; McGill University) table.

10.2196/85002 Checklist 1 PRISMA-ScR checklist.

10.2196/85002 Checklist 2 PRISMA-S checklist

## Abbreviations

MCAT: Medical College Admission Test
MeSH: Medical Subject Headings
MMAT: Mixed Methods Appraisal Tool
PIF: professional identity formation
PRISMA: Preferred Reporting Items for Systematic Reviews and Meta-Analyses
PRISMA-S: Preferred Reporting Items for Systematic Reviews and Meta-Analyses Literature Search Extension
PRISMA-ScR: Preferred Reporting Items for Systematic Reviews and Meta-Analyses Extension for Scoping Reviews
