# Supplementary material for: Retuning the Premedical Compass in American Programs Worldwide: Scoping Review
Source: JMIR Med Educ. 2026 Apr 9;12:e85002. doi: 10.2196/85002 (PMC13064960; doi:10.2196/85002)
Supplement: Multimedia Appendix 1 [file mededu-v12-e85002-s001.doc]

**Database: Ovid MEDLINE(R) Epub Ahead of Print and In-Process, In-Data-Review & Other Non-Indexed Citations and Daily <September 17, 2025>**
**Search Strategy:**
**1**  Education, Premedical/ (810)
**2**  Students, Premedical/ (233)
**3**  Schools, Medical/ (28974)
**4**  School Admission Criteria/ (5856)
**5**  3 and 4 (1580)
**6**  College Admission Test/ (724)
**7**  3 and 6 (303)
**8**  (medical adj1 prop?edeutic?).mp. (8)
**9**  ((curriculum or course*) adj2 (premedical or premedicine or pre-medical or pre-medicine)).mp. (102)
**10**  ((premedical or premedicine or pre-medical or pre-medicine) adj2 (requir* or education or admission* or accept* or student*)).mp. (1291)
**11**  ((prerequisite* or pre-requisite* or precondition* or pre-condition* or admission or applicant* or apply or applying) adj2 (medical or medicine) adj2 (school* or facult*)).mp. (1023)
**12**  (requir* adj2 (entrance or acceptance) adj2 (medical or medicine) adj2 (school* or facult*)).mp. (2)
**13**  ((prerequisite* or pre-requisite* or precondition* or pre-condition* or applicant*) adj2 (medical or medicine)).mp. (952)
**14**  (admission adj2 (medical or medicine) adj2 (school* or facult*)).mp. (429)
**15**  1 or 2 or 5 or 7 or 8 or 9 or 10 or 11 or 12 or 13 or 14 (3938)
**16**  limit 82 to yr="2000 -Current" (2496)
