# Supplementary material for: Retuning the Premedical Compass in American Programs Worldwide: Scoping Review
Source: JMIR Med Educ. 2026 Apr 9;12:e85002. doi: 10.2196/85002 (PMC13064960; doi:10.2196/85002)
Supplement: Multimedia Appendix 2 [file mededu-v12-e85002-s002.docx]

Embase

Session Results

#11. #10 AND [2000-2024]/py

#10. #1 OR #2 OR #3 OR #4 OR #5 OR #6 OR #7 OR #8 OR #9

#9. (admission NEAR/2 (medical OR medicine) NEAR/2 (school* OR facult*)):ti,ab,kw

#8. ((prerequisite* OR 'pre requisite*' OR precondition* OR 'pre condition*' OR applicant*)

NEAR/2 (medical OR medicine)):ti,ab,kw

#7. (requir* NEAR/2 (entrance OR acceptance) NEAR/2 (medical OR medicine) NEAR/2 (school* OR

facult*)):ti,ab,kw

#6. ((prerequisite* OR 'pre requisite*' OR precondition* OR 'pre condition*' OR admission OR

applicant* OR apply OR applying) NEAR/2 (medical OR medicine) NEAR/2 (school* OR

facult*)):ti,ab,kw

#5. ((premedical OR premedicine OR 'pre medical' OR 'pre medicine') NEAR/2 (requir* OR education OR

admission* OR accept* OR student*)):ti,ab,kw

#4. ((curriculum OR course*) NEAR/2 (premedical OR premedicine OR 'pre medical' OR 'pre

medicine')):ti,ab,kw

#3. (medical NEAR/1 prop$edeutic$):ti,ab,kw

#2. 'school admission'/de AND 'medical school'/de

#1. 'premedical student'/de

.......................................................
