# Supplementary material for: Retuning the Premedical Compass in American Programs Worldwide: Scoping Review
Source: JMIR Med Educ. 2026 Apr 9;12:e85002. doi: 10.2196/85002 (PMC13064960; doi:10.2196/85002)
Supplement: Multimedia Appendix 3 [file mededu-v12-e85002-s003.docx]

|  |
| --- |
| **Search:**  (((DE "Required courses" OR DE "College admission" OR DE "College applicants" OR DE "Admission criteria" OR DE "College entrance examinations" OR DE "Prerequisites" OR DE "College credits" OR DE "College curriculum") AND (DE "MEDICAL schools")) OR DE "Premedical Students" OR (TI (medical N0 prop#edeutic*) OR AB (medical N0 prop#edeutic*) OR KW (medical N0 prop#edeutic*)) OR (TI (((curriculum or course*) N1 (premedical or premedicine or pre-medical or pre-medicine))) OR AB (((curriculum or course*) N1 (premedical or premedicine or pre-medical or pre-medicine))) OR KW (((curriculum or course*) N1 (premedical or premedicine or pre-medical or pre-medicine)))) OR (TI (((premedical or premedicine or pre-medical or pre-medicine) N1 (requir* or education or admission* or accept* or student*))) OR AB (((premedical or premedicine or pre-medical or pre-medicine) N1 (requir* or education or admission* or accept* or student*))) OR KW (((premedical or premedicine or pre-medical or pre-medicine) N1 (requir* or education or admission* or accept* or student*)))) OR (TI ((requir* N1 (entrance or acceptance) N1 (medical or medicine) N1 (school* or facult*))) OR AB ((requir* N1 (entrance or acceptance) N1 (medical or medicine) N1 (school* or facult*))) OR KW ((requir* N1 (entrance or acceptance) N1 (medical or medicine) N1 (school* or facult*)))) OR (TI (((prerequisite* or pre-requisite* or precondition* or pre-condition* or applicant*) N1 (medical or medicine))) OR AB (((prerequisite* or pre-requisite* or precondition* or pre-condition* or applicant*) N1 (medical or medicine))) OR KW (((prerequisite* or pre-requisite* or precondition* or pre-condition* or applicant*) N1 (medical or medicine)))) OR (TI ((admission N1 (medical or medicine) N1 (school* or facult*))) OR AB ((admission N1 (medical or medicine) N1 (school* or facult*))) OR KW ((admission N1 (medical or medicine) N1 (school* or facult*)))) OR (TI (((prerequisite* or pre-requisite* or precondition* or pre-condition* or admission or applicant* or apply or applying) N1 (medical or medicine) N1 (school* or facult*))) OR AB (((prerequisite* or pre-requisite* or precondition* or pre-condition* or admission or applicant* or apply or applying) N1 (medical or medicine) N1 (school* or facult*))) OR KW (((prerequisite* or pre-requisite* or precondition* or pre-condition* or admission or applicant* or apply or applying) N1 (medical or medicine) N1 (school* or facult*)))) |
