# Supplementary material for: Retuning the Premedical Compass in American Programs Worldwide: Scoping Review
Source: JMIR Med Educ. 2026 Apr 9;12:e85002. doi: 10.2196/85002 (PMC13064960; doi:10.2196/85002)
Supplement: Multimedia Appendix 5 [file mededu-v12-e85002-s005.pdf]

Study identification					Study characteristics						
Article ID	Author(S)	Year	Title	Journal	Study type/ design (qualitative/ quantitative...)	Study objective	Eligible for MMAT	Type of study study in MMAT (1, 2, 3, 4, 5)	Start and end dates	Location	Institution(s) involved (university/ program)
**Add courses (25)**											
1	Kim Rheinlander, Dorothy Wallace	2011	**Calculus, Biology And Medicine: A Case Study In Quantitative Literacy For Science Students**	Digital Commons University Of South Florida (Usf)	Case study using mixed methods: quantitative attitudes survey, open-ended post questionnaire, classroom observation.	Evaluate the effectiveness of a course that can integrates necessary mathematical concepts into the premedical / biology curriculum	Yes	5	N/a	Us	Darmouth college
2	Courtney M. Smalley, Vaughn Browne, Bonnie Kaplan, Brian Russ, Juliana Wilson, Resa E. Lewiss	2016	**A Course For Pre-Medical Professions Students Using Point-Of-Care Ultrasound**	Journal Of Ultrasound In Medicine	Quantitative : a survey filled by students to evaluate individual speakers, course content, activities, and facilities.	(1) To use point-of-care ultrasound to highlight educational concepts such as the flipped classroom, simulation, hands-on interaction, and medical exposure; (2) to work collaboratively with peers; and (3) to expose premedical students to mentoring for the medical school application process.	Yes	4		Us	University hospital facilities
3	Rachel Conrad Bracken, Ajay Major, Aleena Paul, Kirsten Ostherr	2021	**Reflective Writing About Near-Peer Blogs: A Novel Method For Introducing The Medical Humanities In Premedical Education**	Journal Of Medical Humanities	Qualitative analysis of one hundred seventy six reflective essays by baccalaureate premedical students written in response to medical student-authored narrative blog posts.	Evaluate the pedagogical utility of medical student blogs for introducing core themes in the medical humanities	Yes	1	August 2016 to april 2018	Us	
4	Joanne Tran,1 Leo Meller,1 Vy Le,1 Jasmine Tam,1 Andrea Nicholas,	2024	**Behavioral Assessment Of Soft Skill Development In A Highly Structured Pre-Health Biology Course For Undergraduates**	Journal Of Microbiology And Biology Education	Quantitative: using a mixed-effect linear regression analysis with interactions for student and group assignments+ average of pre and post quizzes scores	Assess the n120 keystone course series for the human biology major at the university of california, irvine	Yes	4	2021–2022 Academic year, where courses in the fall and spring quarters were conducted in person and the winter quarter was a mixture of in person and [illegible]	Us	University of california, irvine,
60	Claudia Da Silva Carvalho	2024	**Knowledge and Perception of Antibiotic Resistance and Stewardship among Pre-Health and Agriculture Undergraduate Students**	Journal of Microbiology & Biology Education	Mixed: survey	The study aimed to compare knowledge and perceptions of antibiotic resistance between Biology/pre-health and Agriculture undergraduates	Yes	5	2019–2020 academic year	US	Fort Hays State University
5	Gia Merlo	2021	**Mpro: A Professionalism Curriculum To Enhance The Professional Identity Formation Of University Premedical Students**	Medical Education Online	Qualitative: weekly reflection on topics discussed in the course and their shadowing experiences.	Survey changes in students' knowledge and perceptions of the curriculum after completing mpro	Yes	1	2015	Us	Rice university
6	Hansuong Vongsachang, Sachi Oshima, Christine Nguyen, Suzanne Gaulocher, N. Ewen Wang;	2020	**Undergraduate Premedical Student Perceptions Of An Emergency Department–Based Social Needs Screening Program**	Medical Science Educator	Mixed : assess student perceptions of program impact through student applications, course evaluations, and an online program survey.	Understand the impact of social science based, experiential learning programs on premedical students' education and preparation for medical [illegible]	Yes	5	N/a	Us	Stanford university
7	Jason Wilson	2019	**Patient Shadowing: A Useful Research Method, Teaching Tool, And Approach To Student Professional Development For Premedical Undergraduates**	Academic Medicine	Qualitative: thematic analysis of students' field notes and reflection essays and follow up communications with course alumni .	Evaluate a course that provides opportunities for patient and physician shadowing within the anthropological methodological framework of participant [illegible]	Yes	1	2016 Till 2018	Us	University of south florida, tampa, florida
8	Joseph P. Forester, David L. Mcwhorter,* And Maria S. Cole;	2002	**The Relationship Between Premedical Coursework In Gross Anatomy And Histology And Medical School Performance In Gross Anatomy And Histology**	Clinical Anatomy	Quantitative: survey of students scores	Determine whether a premedical gross anatomy and/or histology course resulted in increased performance in corresponding medical school courses and examine whether the type of premedical gross anatomy and/or histology course differentially affected medical school performance	Yes	4	N/a	Us	University of health sciences college of os[illegible] city, missouri,
9	Lauren D. Olsen	2016	**"It'S On The Mcat For A Reason": Premedical Students And The Perceived Utility Of Sociology**	American Sociological Association	Qualitative: analysis of 490 reflective student journal entries using N-Vivo	Examine how premedical students enrolled in an introductory sociology course perceive the value of sociology for medical practice	Yes	1	Na	Us	Na
10	Malvitz, Madelyn; Khan, Noreen; Morgenstern, Lewis B	2024	**College Preparation For A Medical Career In The United States.**	Plos One	A collective case series framework was used to obtain qualitative data.	Consider the mismatch between student needs in health science and available resources in colleges across the united states	Yes	1	June 6, 2022 - july 21, 2022	Us	20 Colleges
11	Michael F. Antolin, Kristin P. Jenkins, Carl T. Bergstrom, Bernard J. Crespi, Subhajyoti De, Angela Hancock, Kathryn A. Hanley, Thomas R. Meagher, Andres Moreno-Estrada, Randolph M. Nesse, Gilbert S. Omenn, And Stephen C. [illegible]	2011	**Evolution And Medicine In Undergraduate Education: A Prescription For All Biology Students**	Outlook On Evolution And Society	Not a study.		No				
12	Milos Savic,Paxtonmartin;	2018	**The Perceived Versus Actual Use Of Mathematics In Medicine According To Pre-Medicine Students And Practicing Physician**	Oxford University Press	Qualitative : interviews were carried out with a semi-structured interview with both predetermined and impromptu questions, mostly to allow the participant to clarify their previous statements.	Assist universities trying to unify their mathematics requirements for premed majors	Yes	1	N/a	Us	University of oklahoma, norman, oklahom[illegible]
13	Natalie K. Cooke, Sarah L. Ash, L. Suzanne Goodell And Kelsey L. Wilson	2015	**Qualitative Assessment Of Pre-Healthcare Undergraduates' Perceptions Of Childhood Obesity To Inform Premedical Curricular Changes**	North American Colleges And Teachers Of Agriculture (Nacta)	Qualitative: interviews were conducted to collect data for this study.	Examine to what degree prehealthcare undergraduates can identify and describe the complexity of childhood obesity to inform premedical curricular approaches in light of the 2015 changes to the medical college [illegible]	Yes	1	2015	Us	North carolina state university.
14	Peter Kondrashov	2017	**Premedical Anatomy Experience And Student Performance In Medical Gross Anatomy**	Medical And Dental Education	Mixed: qualitative survey responses (google form) were summarized as number of responses and percentage.	Evaluate the effect of having taken an anatomy course prior to entering osteopathic medical school on performance in the gross anatomy course during the first year of [illegible]	Yes	5	2017 Till 2019	Us	Kirksville college of osteopathic medicine
15	Randolph M. Nesse	2010	**Making Evolutionary Biology A Basic Science For Medicine**	The Proceedings Of The National Academy Of Sciences (Pnas)	Not a study.		No		Na	Us	Na
16	Rita Charon	2000	**Reading, Writing, And Doctoring: Literature And Medicine**	The American Journal Of The Medical Sciences	Not a study.		No			Us	
17	Shilpa J. Buch	2011	**Neuroimmune Pharmacology As An Emerging Curriculum For Pre-Medical Students**	Neuroimmune Pharmacology	Not a study.		No		Na	Us	Na
18	Sue Ann Miller,* William Perrotti, Dee U. Silverthorn, Arthur F. Dalley, And Kyle E. Rarey	2002	**From College To Clinic: Reasoning Over Memorization Is Key For Understanding Anatomy**	The Anatomical Record	Not a study.		No			Us	
19	Vijaya B. Kola Chalama	2022	**Machine Learning And Pre-Medical Education**	Artificial Intelligence In Medicine	Not a study.		No		Na	Us	Na
59	Stevens, Courtney; Witkow, Melissa R	2014	**Training scientific thinking skills: Evidence from an MCAT2015-aligned classroom module**	Teaching of Psychology	Quantitative: Two of the sections received a scientific thinking module (45 students, one section	The present study reports on the development and evaluation of a classroom module to train scientific	Yes	4	Fall 2013/early 2014	US	Willamette University
67	Afghani B.; Manalang J.; Salazar A.; Lee J.; Kim K.	2013	**Should cadaver lab be introduced to premedical students in an effort to promote their interest towards health careers?**	Journal of Investigative Medicine (Western Regional Meeting Abstracts)	Quantitative : survey-based evaluation.	To determine whether cadaver lab exposure increases interest in health careers among high school and college [illegible]	Yes	4	Summers of 2010–2012 (2-week summer program)	Us	University of California, Irvine School of [illegible]
20	Marlene D. Louro, Grace Meegan, Lauren R. Rudin, Nathan E. Thompson	2023	**Students With Prior Anatomy Experience Start Out Stronger In Medical School Gross Anatomy**	American Association For Anatomy	Quantitative: survey students from all four matriculated years and analyzed their individual anatomy laboratory exam scores and final anatomy laboratory course scores.	Investigate the relationship between pre-medical school anatomy experience and medical school gross anatomy performance among students at new york institute of technology, college of osteopathic [illegible]	Yes	4	October and december 2022	Us	New york institute of technology, college [illegible]
21	Raddy L. Ramos, Erik Guercio, And Luis R. Martinez	2017	**Pre-Medical Preparation In Microbiology Among Applicants And Matriculants In Osteopathic Medical School In The United States**	Journal Of Microbiology & Biology Education	Not a study.		No		2012 Till 2016	Us	The american association of colleges of os[illegible] manages a centralized application system [illegible] medical schools exception of university o[illegible] of osteopathic medicine (unthsc-tcom) an[illegible] technology college of osteopathic medicin[illegible]
22	Rebecca Thomas, Taylor Yancey, Chad Skidmore, Neal Ferrin, Iain Zapata, Jennifer Williams, Nena Lundgreen Mason	2021	**The Effects Of Pre-Medical Anatomy And Clinical Experiences On Medical School Anatomy-Related Academic Performance**	Medical Science Educator	Quantitative: survey to assess pre-medical clinical and anatomical experiences and results were compared to each student's course grades and anatomy practical	Examine the effects that pre-medical patient care clinical experiences and anatomical education had on undergraduate medical course grades and anatomy practical exam	Yes	1	Between academic years 2010–2011 and 2018–2019	Us	Rocky vista university col lege of osteopa[illegible]
**Add+ remove + redesign courses (1)**											
23	Alpern, R.J.; Belitsky, R.; Long, S.	2011	**Competencies In Premedical And Medical Education**	Perspectives In Biology And Medicine	Not a study but rather a summary of the hhmi aamc report findings		No			Us	
**Add + remove courses (4)**											
24	Cahn	2014	**Biologic: Gene Circuits And Feedback In An Introductory Physics Sequence For Biology And Premedical Students**	American Journal Of Physics			No		2010-2013	Us	Yale university
25	David Muller, And Nathan Kase	2010	**Challenging Traditional Premedical Requirements As Predictors Of Success In Medical School: The Mount Sinai School Of Medicine Humanities And Medicine Program**	Academic Medicine	Quantitative: cross-tabulations andanovacomparing humedstudentswith non-humed students across various database variables.	Assess the medical school performance of humanities and social science majors who omitted organic chemistry, physics, and calculus, and did not take the mcat.	Yes	4	6 Years: graduating class of 2004-2009	Us	Mount sinai school of medicine
26	Wershof Schwartz, Andrea, Abramson, Jeremy S, Wojnowich, Israel, Accordino, Robert, Ronan, Edward J, Rifkin, Mary R	2009	**Evaluating The Impact Of The Humanities In Medical Education.**	The Mount Sinai Journal Of Medicine	This article reviews the literature on the impact of humanities education on the performance of medical students.		No			Us	Mount sinai school of medicine
27	William W. Kennerly, Kimberley A. Frederick, And Kelly Sheppard	2020	**General Chemistry In Just One Semester For All Majors**	Journal Of Chemical Education	Quantitative: assess the impact on learning development of one semester general chemistry with the colorado learning attitudes about science survey chemistry version [illegible]	Evaluate the impact of a one semester general chemistry course (instead of 1 year)	Yes	4	Na	Us, new york	Skidmore
**Add + redesign courses (6)**											
28	Chambers, Donald A, Cohen, Rhonna L, Girotti, Jorge	2011	**A Century Of Premedical Education.**	Perspectives In Biology And Medicine	Not a study: a historical overview		No		1910-2011	Us	University of illinois at chicago
29	David R. Haburchak, Bradford C. Mitchell, And Craig J. Boomer	2008	**(Re-) Defining Evolutionary Medicine**	Philosophy Of Medical Practice			No		Na	Us	Na
30	Ferren, Ann S, Ferren, Peter M	2012	**Faculty Leadership In Premedical Education**	Proquest			No				
31	Pablo Rodriguez Del Pozo, And Joseph J. Fins	2005	**The Globalization Of Education In Medical Ethics And Humanities: Evolving Pedagogy At Weill Cornell Medical College In Qatar**	Academic Medicine	Mixed : students feedback and rating of the course	Discuss their experience in implementing a medical ethics and humanities course for premedical students at weill cornell medical college in the arabian gulf [illegible]	Yes	5	N/a	Qatar	Weill cornell medical college
32	Karen Mitchell, Jason Satterfield, Richard S. Lewis, Barry A. Hong	2016	**The New Medical College Admission Test: Implications For Teaching Psychology**	The American Psychologist			No		N/a	Us	N/a
33	Tyler J Stodola And Sean P Bruna	2021	**Medical Anthropology Courses And Concepts Tested On The Mcat: A Content Analysis Of 40 U.S. Course Syllabi**	Journal Of Medical Education And Curricular Development	Mixed methods content analysis, survey data examined using descriptive statistics; syllabi were analyzed with nvivo using a binary coding scheme and modified grounded theory	Examine if anthropological coursework addresses relevant mcat topics	Yes	5	Na	Us	Western washington university
**Redesign courses (18)**											
34	Cathryn A. Peterson, Richard P. Tucker	2005	**Undergraduate Coursework In Anatomy As A Predictor Of Performance: Comparison Between Students Taking A Medical Gross Anatomy Course Of Average Length And A Course Shortened By Curriculum Reform**	Clinical Anatomy	Quantitative: investigate the relationship between undergraduate anatomy coursework and performance in medical gross anatomy using class rank and final exam scores	Evaluate whether taking undergraduate anatomy (type, amount, and grades) improves performance in a traditional vs. Shortened medical anatomy course	Yes	4	1992-2002	Us	University of califor nia at davis
63	Christian Moro; Michelle McLean; Charlotte Phelps	2025	**Doing justice when incorporating sustainability into pre-medical curricula**	Medical Education	Mixed: educational intervention + survey feedback analysis	To trial a low-burden approach to embedding Planetary Health into pre-medical physiology lectures and assess student [illegible]	Yes	5	Semester-long intervention: 12 weeks; Received Oct 2024 – Accepted Feb 2025 – Published 2025	Bond University, Queensland, Australia	Faculty of Health Sciences & Medicine, B[illegible]
35	Kathryn C. Bolduc, ShannonI. Mccollough , AngelaK.Stoeckman	2022	**From Classroom To Clinic: Biochemistry Lab For Pre-Health Majors**	Biochemistry And Molecular Biology Education.	Quantitative: pre and post class survey (likert scale responses, % of students reporting gains)	Evaluate whether a biochemistry lab module increases understanding of clinical lab techniques among pre-health students	Yes	4		Us	Bethel university

	Participants		Premedical course(s) discussed		Changes to the courses							
Article ID	Population described (students, educators, admissions officers, etc.)	Characteristics of participants (number of participants, major...)	Type of courses (number of credits, lab or non lab...)	Nature of change (at curriculum or course level, number of courses)	New course(s) introduced	Existing course(s) removed	Content of existing course(s) revised	Drivers of change (aamc guidelines, institutional decisions...)	Outcomes	Comments from study authors/ remarks	Themes/importance	Common outcomes
1	Students and educators	The majority of students enrolled in math 4 are planning a career in medicine. Many but not all of these are majoring in biology. Typically the class has 15-20 students.	Non-lab	Addition of math and biology courses.	The course applications of calculus to medicine and biology bridges abstract mathematical concepts with real-world biomedical and ecological problems, aiming to help biology and premedical students understand dynamical systems through phase portraits and applied modeling.			Since 2003, wallace has taught a course for biology and pre-medical students that builds on introductory calculus to model changes in biological quantities.	Strong improvement in student attitudes about math.	For these students mathematics is about learning more math content and not how to apply the math they know in a creative, integrated and precise way.	Add math (biology and math)/ important	
2	Students enrolled in the course	Thirteen women and 2 men	Point-of-care ultrasound course for premedical students	Addition of a course about point-of-care-ultrasound.	This course uses point-of-care ultrasound to teach anatomy through hands-on, peer-led learning. It combines clinical exposure, physician shadowing, and med school application prep, helping students build skills and confidence early on.			Point-of-care ultrasound (pocus) is now a key skill in medical training. It's become a national standard in emergency medicine and needs to be increasingly included in undergrad med school curricula.	Students with no prior ultrasound experience became confident in basic point-of-care ultrasound. Small number of students (15 students) still showed strong outcomes.	Barriers like needing access to equipment, trained ultrasound faculty, and strong ties between undergrad and med school programs can limit replication. Securing proper spaces or venues is another challenge.	Adding a new course (point-of-care-ultrasound)/ important	
3	62 Premedical students	[illegible]	[illegible]	[illegible]	[illegible]	[illegible]	[illegible]	[illegible]	[illegible]	[illegible]	[illegible]	[illegible]
4	Students	[illegible]	[illegible]	[illegible]	[illegible]	[illegible]	[illegible]	[illegible]	[illegible]	[illegible]	[illegible]	[illegible]
60	undergraduate students majoring in Biology/pre health and Agriculture	[illegible]	[illegible]	[illegible]	[illegible]	[illegible]	[illegible]	[illegible]	[illegible]	[illegible]	[illegible]	[illegible]
5	Premedical students and faculty and physicians	[illegible]	[illegible]	[illegible]	[illegible]	[illegible]	[illegible]	[illegible]	[illegible]	[illegible]	[illegible]	[illegible]
6–35	[illegible]	[illegible]	[illegible]	[illegible]	[illegible]	[illegible]	[illegible]	[illegible]	[illegible]	[illegible]	[illegible]	[illegible]

36	David M. Harris, Christine Bellew, Zixi J. Cheng, Juan C. Cendán,And Jonathan D. Kibble	2014	**High-Fidelity Patient Simulators To Expose Undergraduate Students To The Clinical Relevance Of Physiology Concepts**	Advanced Physiological Education	Quantitative: pre-post test design measuring physiology knowledge and student feedback	Assess feasibility, student learning, and engagement through a simulation-based physiology activity using high-fidelity patient simulators (hfps)	Yes	4		Us, orlando, florida	University of central florida college of medicine, orlando, florida	Biomedical students in our under graduate program		Physiology class+ lab	Physiology course			The 2-hour high-fidelity patient simulation (hfps) session was part of an upper-level undergraduate course called "laboratory virtual simulations in physiology." The course is delivered in a mixed format (both online and limited in-person sessions).	This article argues that giving undergrads, especially future healthcare workers, chances to see physiology in real-life contexts can boost learning, interest, and career direction. With rising healthcare demands, such activities may encourage more students to pursue health and stem careers.	Improvement was statistically significant (p = 0.005, Paired student's t-test). The simulation helped students connect class-taught physiology to clinical practice, making the learning more meaningful and relevant.	Future studies are necessary to assess longer-term learning retention.	Revise laboratory virtual simulations in physiology/ important	
37	Jon-Philippe K Hyatt & Stephen A Hurst	2010	**Novel Undergraduate Physiology Laboratory Using A Human Patient Simulator**	Medical Education	Qualitative: descriptive report of a lab-based pedagogical approach, including student reflections and instructor	Apply physiology knowledge through creative, student-designed experiments	Yes	1	Na	Us	Na	Students		Non lab	"Physiological adaptation" course			The meti human patient simulator (hps) is used in the "physiological adaptation" course to explore human adaptation to environmental extremes rather than disease.	Undergraduate pre-medical education is generally taught using traditional didactic laboratories that include stepwise methodologies and expected outcomes. A human patient simulator (hps) can be used as an open-ended laboratory tool outside the clinical spectrum to immerse undergraduates in scientific inquiry.	Course evaluations reveal positive reviews of this laboratory experience because it allows the opportunity to investigate topics in greater depth than in earlier coursework.	Students have expressed some frustration that the hps data were not always in line with their predicted outcomes. This sentiment also reflects, in part, the inherent limitations of the hps since it cannot replicate	Redesigning the 'physiological adaptation' course/ important	
38	Moebrie, S.G.J.	2016	**Vision And Change In Introductory Physics For The Life Sciences**	American Journal Of Physics	Mixed: includes quantitative student evaluations (e.G. Likert-scale surveys, sets) and qualitative feedback (open ended student quotes)	Assess how a reformed, biologically-relevant physics course affects student perception of physics' relevance to biology/medicine and how it compares to traditional courses	Yes	5	2006-2014	Us	Unspecified	Students	Students enrolled in the reformed physics course	Both lab and non-lab	Course level			Reports have emphasized the growing need for strong quantitative skills among undergraduate students pursuing biomedical careers, prompting calls to reform premedical and biology education. Frameworks like vcube and sfp outline core competencies—many aligned with physics education—positioning the introductory physics for the life sciences (ipls) sequence as a key opportunity to introduce students to quantitative and mathematical modeling of biological systems.	Quantitative skills are increasingly vital for biomedical careers, prompting reforms in undergraduate biology and premedical education. The ipls physics sequence offers a key opportunity to build these skills through biologically relevant applications.	In comparison to traditional first-semester introductory physics, biological science majors and premedical students give a comparable or higher overall rating (general physics or university physics, respectively) to a version of first-semester introductory physics (university physics for the life sciences) that significantly adjusts the introductory physics syllabus to incorporate a selection of topics that are much more biologically and medically relevant.	It is difficult to see how to address this problem at the departmental level, a related problem would be implementation.	Revise physics / important	
39	Sherri C. Young	2016	**Comparable Educational Benefits In Half The Time: An Alternating Organic Chemistry Laboratory Sequence Targeting Prehealth Students**	Journal Of Chemical Education	Mixed: quantitative assessment (exam scores, report grades, lab practical's) and qualitative feedback (student surveys and comments)	Assess whether an revised lab model offers equivalent educational outcomes to traditional labs for prehealth students	Yes	5	2012-2015	Us	Muhlenberg college	Students	Student enrollments all three years were typical of our department (70−80 students in the fall and 50−65 students in the spring). It should be noted that chemistry and biochemistry majors are advised to take a separate majors organic course; therefore, the students enrolled in this course were predominantly biology, neuroscience, and natural science majors, and a majority of these students were also prehealth.	Lab	Cours level			At muhlenberg college, the organic chemistry laboratory curriculum was revised to dedicate more student time to process skills, which transcend chemistry and are useful in any discipline.	The scientific foundations for future physicians (sffp), a report addressing global concerns about education in premedical and medical school programs.	In general, student performance, measured by laboratory report grades, prelab quiz grades, lab note book grades,90 min lecture exam grades, fall-semester final exam grades, and acs organic chemistry exam grades (administered each spring), was consistent between our no alternating (2012−13) and alternating lab sequences (2013−14/2014−15),	Science, technology, engineering, and math (stem) education research suggests that students learn best when they are actively engaged with the material at hand. This new lab model, in which almost all of the work is inquiry-based, allows students to take ownership of their learning by constructing their own explanations for key chemistry phenomena, which enhances learning. Furthermore, many students work in groups on the alternate-week exercises; evidence suggests that collaboration can improve learning and retention.	Revise chemistry lab/ important	Positive feedback about the course.
62	Jessica Watkins; Janet E. Coffey; Edward F. Redish; Todd J. Cooke	2012	**Disciplinary authenticity: Enriching the reforms of introductory physics courses for life-science students**	Physical Review Special Topics – Physics Education Research	Qualitative / Descriptive: theoretical framework, task analysis, student interviews	Expand the construct of disciplinary authenticity; examine how an introductory biology course uses math/physics and how students respond	Yes	1	Spring 2010 & Fall 2010 courses; published Apr 2012	University of Maryland, USA	Dept. of Physics; Dept. of Curriculum & Instruction; Dept. of Cell Biology & Molecular Genetics	Undergraduate biology students and instructors	Spring N=147; Fall N=80; classroom observations; student interviews (2 detailed cases)	Introductory Organismal Biology (lecture + group work, non-lab)	Course-level reform integrating math/physics into biology			Revised content of existing biology course (integration of physics/math concepts)	Active-learning worksheets using Fick's law, diffusion, levers; qualitative reasoning with equations	Students applied physics/math tools to biological problems; demonstrated varied engagement/authenticity	Authors highlight "disciplinary authenticity" as key to interdisciplinary integration	Redesign courses (biology)	
64	Michael C. Lotspeich-Yadao; Kimberly Trevino-Boissel; Cara Hoekstra	2025	**Integrating Experiential Learning in Introduction to Sociology: Cultivating the Sociological Imagination in Premedicine Students**	Teaching Sociology	Mixed: pre/post surveys, reflective journals, grounded theory	To examine the role of experiential learning in an Intro to Sociology course for premedicine students, especially in cultivating sociological imagination and understanding health disparities	Yes	5	Fall 2018 semester (CHNA project timeline)	Baylor University, Waco, Texas, USA	University of Illinois Urbana-Champaign; Baylor University	Premedical undergraduates in Introduction to Sociology	N=55 enrolled; 49.1% premedicine; diverse majors (biology, pre-business, prenursing, etc.); majority female (67%); racially diverse (65% White, 20% Hispanic, 11% Asian, 2% Black)	SOC 1305 – Intro to Sociology; lecture + flipped classroom + experiential project	Course-level reform (integration of applied research into existing course)			Revised existing Intro Sociology course to embed experiential research with community health	15 hours applied research: 9 hours phone surveys, 6 hours door-to-door interviews; integration of reflection journals; flipped format for lectures	Statistically significant improvements in writing, empathy, understanding local community; modest increases in critical thinking and diversity appreciation.	MCAT 2015 changes emphasizing sociology/social determinants	Redesign courses (sociology)	
65	Catherine H. Crouch; Kenneth Heller	2014	**Introductory physics in biological context: An approach to improve introductory physics for life science students**	American Journal of Physics	Not a study	To restructure introductory physics courses to emphasize biologically authentic contexts, develop quantitative/problem-solving skills, and improve relevance for life	No		Implemented from 2008 onward; article received Aug 2013, accepted Mar 2014, published May 2014	Swarthmore College (PA), University of Minnesota (MN),	Dept. of Physics & Astronomy, Swarthmore College; School of Physics & Astronomy, University of Minnesota	Undergraduate life science and premedical students	Minnesota: ~120–200 per section, 3 concurrent sections; Swarthmore: ~90–120 students; biology, chemistry, premed majors	Introductory Physics for Life Sciences (IPLS), calculus-based; lecture + lab + discussion	Major course-level reform (scope, sequence, pedagogy)			Removed or reduced certain physics topics less relevant to biology (e.g., momentum, angular dynamics, Gauss's Law, AC circuits)	Added/revised content to focus on fluid dynamics, diffusion, osmosis, optics, electrostatics in biological media, energy/entropy in open systems; integrated biological examples	Improved student attitudes toward physics (CLASS survey); better conceptual gains on FCI & BEMA compared to traditional courses; increased appreciation of physics relevance for biology	Shows feasibility of IPLS reform across different institutions; importance of authentic biological contexts; cognitive apprenticeship framework	Redesign courses (physics)	
40	Shawn K. Stover, Davis & Elkins College	2020	**Integrating Case Studies Into A Pre-Medical Curriculum**	Bio Scene, Association Of College & University Biology Educators			No			Us	Davis & elkins college (d&e)			Course including lecture and lab	Course level for 3 courses			Clinical cases were added to a 100-level physiology, 200-level histology, and 300-level biochemistry course. Students analyzed directed case studies to build critical thinking. Cases were done in labs, lectures, or as homework, depending on the course. This method helped	Group case studies build critical thinking, make content feel more relevant, and help students learn from different perspectives.	These methods help reinforce content, stimulate engagement, and prepare students for clinical reasoning.		Revise a 100-level physiology course, a 200-level histology course, and a 300-level biochemistry course/ important	
41	E. F. Redish	2014	**Nexus/Physics: An Interdisciplinary Repurposing Of Physics For Biologists**	American Association Of Physics Teachers	Mixed: qualitative observations, classroom videos, interviews, and quantitative pre/post	Evaluate a restructured physics curriculum	Yes	5	2011–2012 And 2012–2013 and 2013–2014	Us	University of maryland, purdue university, umbc, university of miami	Premedical students	The ccb grant brings together disciplinary education specialists in physics, biology, and chemistry from six universities (university of maryland, university of colorado, university of new hampshire, swarthmore college, michigan	Lab and nonlab	Introductory physics courses			Nexus/physics is a second-year physics course for life-science majors that bridges biology, chemistry, and physics. It focuses on relevant topics like energy, fluids, thermodynamics, and random motion at cellular and	Traditionally, physics courses have not addressed the specific needs of life-science students, often offering only superficial biological context. Nexus/physics challenges this approach.	It is possible for biology majors to see learning physics as rewarding, engaging, and valuable to their future scientific pursuits.	The nexus/physics course has gone through three years of discussion, development, and testing.	Redesigning a physics course/ very important	Positive impact and improvement for future career development/ med school.
68	Kavitha T. Ram; Howard A. Blanchette	2015	**How Should We Teach Physicians the Language of Statistics?**	Obstetrics & Gynecology (CREOG & APGO Supplement Abstracts)	Quantitative: national survey of medical school deans	To explore when statistics training should begin (premedical vs. medical years), optimal duration, and impact on	Yes	4	Survey conducted 2014; published Oct 2015	United States	New York Medical College	U.S. medical school deans	N=100 deans responded (40.3% response rate out of 248 invited)	Undergraduate (premedical) statistics; medical school preclinical statistics	Curriculum-level recommendation (undergraduate + medical school integration)			Recommended revising requirements to include statistics at premed and med levels	One semester premedical + one semester medical school statistics proposed; premade exposure linked to higher research productivity	71% deans believed statistics should start at undergraduate premedical level; 2 semesters most efficient for fluency	Early statistics training improves research productivity; lack of consensus on exact implementation	Redesign courses (statistics)	
42	Robert C. Hilborn	2014	**Physics And The Revised Medical College Admission Test**	American Journal Of Physics			No		Na	Us	Na	Premedical students, medical school faculty, medical students, and medical school admis sion staff	Na	Physics courses and the associated instructional laboratories	Course level (introductoryphysicsforthelife sciences(ipls))			Intro physics for life science students should cover transport processes, statistics, and microscopic models, with real biological examples like oxygen diffusion in flatworms. Physiology and neuroscience are key applications, but a standard curriculum and relevant labs are still missing.	The scientific foundations for future physicians (sffp) report outlines key scientific competencies for future doctors, focusing here on undergraduate physics preparation. Competency e3 highlights the need to understand basic physical principles as they apply to living systems. Additionally, competencies e1 and e2 emphasize quantitative reasoning and scientific inquiry.	Redesigning physics courses for life science students means cutting some topics or making courses longer, but longer courses aren't practical. Since students have varying biology knowledge, some biology may need to be taught with physics. Despite challenges, updating these courses is important for health science careers and biophysics growth.	Na	Redesigning physics course/ important	
43	Linnea Dixson, Briana Pomales, Mehrtash Hashemzadeh, And Mehrnoosh Hashemzadeh	2022	**Is Organic Chemistry Helpful For Basic Understanding Of Disease And Medical Education?**	Journal Of Chemical Education	Quantitative: anonymous responses from medical students analyzed with chi-square test	Assess current u.S. Medical students' perspectives on the value of organic chemistry in their premedical education	Yes	4	2020-2021	Us	University of arizona college of medicine phoenix.	Medical and premedical students	566 Medical students enrolled in 46 different medical schools.	Organic chemistry courses	Course level			Organic chemistry should remain in undergraduate curricula because students feel it improves critical thinking. However, teaching methods need re-evaluation, especially for premedical courses. Focusing on foundational topics linked to real-life clinical examples could boost critical thinking, retention, and relevance, potentially reducing dropout rates and negative attitudes toward the course.	While the aamc requires it, its value as a premed prerequisite is debated due to high dropout rates. Studies show many premed students leave the track because of organic chemistry, raising questions about whether one course should have such a significant impact on students' medical career paths.	About 57% of students from public programs and 53% from private programs see organic chemistry as valuable in premed education	The majority of students reported that the critical thinking skills acquired through organic chemistry have proven to be valuable in medical school. But more research is needed to clarify why it remains highly valued by admissions committees.	Redesigning organic chemistry/ important	
44	Gerd Kortemeyer	2007	**The Challenge Of Teaching Introductory Physics To Premedical Students**	The Physics Teacher	Mixed: quantitative surveys (pre/post) and qualitative free-text feedback	Analyze the mismatch between instructor goals and premedical student expectations in physics	Yes	5	N/a	Us	Lyman-briggs college and division of science and mathematics education, michigan state university, east lansing, mi	Students	Na	Non-lab	Change in calculus-based introductory physics course for premedical students			Replacing standard textbook problems with symbolic, representation-translation questions and incorporating medically relevant examples may improve math link outcomes, though grading constraints limit ideal	This article examines the disconnect between physics course goals and mcat requirements, urging reform by exploring student expectations and proposing strategies to better align physics education with premed needs.	Qualitative analysis revealed premeds desire more explicit medical and mcat-related examples in physics courses, while non-premeds resist mcat-focused content, highlighting a disconnect in perceived relevance and a need for more intentional integration of medical applications.		Revise calculus based physics course/ important	New insights about a course.
45	Ryan L. Stowe And Melanie M. Cooper	2017	**Practicing What We Preach: Assessing "Critical Thinking" In Organic Chemistry**	Journal Of Chemical Education	Mixed: analysis of 118 exam items from elite universities using a validated rubric	Assess whether organic chemistry assessments align with the goal of promoting critical thinking and scientific practices	Yes	5	Na	Us	Michigan state university	Premedical students that took an organic chemistry course	Na	Organic chemistry	Course level			Traditional organic chemistry exam questions, including those from elite universities and the acs exam, often test memorization and pattern recognition rather than deep understanding. Students can perform well without truly grasping core concepts. To genuinely assess understanding, exams must prompt students to explain their reasoning.	Organic chemistry is often justified as promoting problem-solving and diagnostic thinking, but these skills are poorly defined and rarely assessed intentionally. Since assessments shape what students focus on, it's essential to evaluate not just content recall but also scientific practices, like modeling, data analysis, and argument from evidence, to truly support meaningful learning and intellectual development.	While organic chemistry often claims to develop problem-solving and scientific thinking, traditional assessments rarely reflect these goals. Since assessments signal what matters in a course, they should actively engage students in scientific and engineering practices (seps).	We are hopeful that the exemplars provided here will serve as a starting point for instructors wishing to assess seps and prompt reflection on meaningful three-dimensional assessments.	Redesigning the exams and not the content/ not important	
46	Elliot Mylott, Ellynne Kutschera, Justin C. Dunlap, Warren Christensen, Ralf Widenhorn	2016	**Using Biomedically Relevant Multimedia Content In An Introductory Physics Course For Life Science And Pre-Health Students**	Journal Of Science Education And Technology	Mixed: combines quantitative survey results with qualitative open-ended responses	Assess the impact of a biomedically-focused, flipped-classroom physics course	Yes	5	April to june 2014	Us	Portland state university (psu)	Students taking the course	Students registered for the course during the spring 2014 semester	Both	Course level			This pilot physics course for pre-health and life science majors used a flipped classroom and biomedical content to make physics more relevant and engaging. It included videos, group work, and applied assignments, receiving positive feedback from students. Offered in spring at psu, it reformed a section of the traditional algebra-based sequence, which spans mechanics, thermodynamics, electromagnetism, and modern physics across the academic year, with an additional biomedical physics elective	Pre-medical education has been criticized for lacking coherence and relevance, prompting calls for reform toward an interdisciplinary, outcome-based, and inquiry-driven approach. With rapid advancements in medical science and technology, a strong foundation in physics, chemistry, and mathematics is increasingly essential for future physicians. However, most algebra-based physics courses, though filled with pre-health students, rarely emphasize biomedical applications.	Unlike traditional algebra-based physics courses, which often mirror calculus-based ones with less math and limited relevance for life science students, the psu course is tailored to biology majors by focusing on biomedical applications and devices.	The small sample size from one iteration of the one quarter pilot course and the selfreported nature of the data make it difficult to draw final conclusions, although these initial results are encouraging.	Revise introductory physics course/ important	
**Proposition of a curriculum (10)**																							
47	Barbara E. Goodman, Karen L. Koster, And David L. Swanson;	2015	**The Development And Implementation Of A New Medical Biology Major Including Physiology**	Advanced Physiology Education			No		Spring semester of 2011	Us	University of south dakota, vermillion, south dakota	Committee	Educators in the college of arts and science and in medical school	Both	Curriculum: add a new major	To better meet sffp competencies, a new medical biology major was launched in 2012 with added inquiry-based, team-focused, and human-centered physiology courses, addressing prior gaps in lab work, critical thinking, and relevance to medical education.		The committee restructured the chemistry and biology curriculum to better align with medical competencies, introducing a flexible medical biology major in 2012 with revised biochemistry, biomolecular, and general education requirements, plus diverse electives taught by medical faculty.	The committee recommended one semester of organic chemistry (expanded from 4 to 5 credit hours) with a laboratory followed by a new one-semester chemistry course about the structure and function of biomolecules without a laboratory. A cell biology course emphasizing the chemistry of biomolecules in cellular function would be allowed as an alternative to the structure and function chemistry course to give a wider range of options and scheduling flexibility to students. These included calculus, general psychology, introduction to sociology, and biomedical ethics courses. As electives within the major, medical biology students can take a number of biology courses or microbiology, pharmacology, physiology, public health, epidemiology, or biochemistry	Suggestion of a new premedical competencies curriculum	.	Design of a new major (new medical biology major)/ important	
48	David R. Haburchak, Bradford C. Mitchell, And Craig J. Boomer	2008	**Quixotic Medicine: Physical And Economic Laws Perilously Disregarded In Health Care And Medical Education**	Philosophy Of Medical Practice			No		Na	Us	Medical college of georgia	Students (premed and med), residents, and faculty		Lab and non lab	Curriculum	Humanities: bible and other scripture, classic literature, history, cultural anthropology, sociology. Sciences and social sciences: physics (at least one semester), macroeconomics (at least one semester), microeconomics (at least one semester), developmental and behavioral psychology. Other fields: principles of management and leadership, ethics, logic, industrial engineering, education, marketing,			The majority of students reported that the critical thinking skills acquired through organic chemistry have proven to be valuable in medical school.	The article proposes eight laws to apply to health care: four physical laws; finitude, inertia, entropy, and uncertainty, and four economic laws; diminishing returns, unintended consequences, distribution, and economizing.	The authors recommend that premedical education return to a broader liberal arts curriculum and that medical education and training foster didactic and experiential knowledge of these eight laws.	Turning the premed curriculum into a broader liberal arts curriculum/ important	
49	Fishbein, R H	2001	**Origins Of Modern Premedical Education.**	Academic Medicine : Journal Of The Association Of American Medical Colleges			No		Primarily late 19th century to early 20th century (1870s–1910s)	Us	The johns hopkins university, harvard medical school, university of michigan, cornell university, university of pennsylvania	Early medical students and premedical students in the united states; administrators and educators shaping premedical education such as daniel coit gilman, john shaw billings, william welch		Lab and non lab	Implementation of the curriculum through history	Proposed curriculum: following is the course of study as described in the 1878 university catalog: first year experimental physics (lectures and laboratory work) elementary mechanics chemistry (lectures and laboratory work) french and german drawing (free hand) second year chemistry (continued) general biology (lectures and laboratory work) elementary course in comparative anatomy and zoology (lectures and dissections) elementary course in physiology and histology (lectures and laboratory work) human osteology and the anatomy of the ligaments and joints third year human anatomy advanced course in physiology and histology elements of embryology psychology logic"			In the late 1800s, johns hopkins prioritized science, research, and lab work over a purely literary approach. This shift made it clear that future med students needed a solid background in physics, chemistry, and physiology even before starting med school.	The quality and rigor of medical education depends heavily on students' academic preparedness. A strong liberal education from a four-year college program is still highly valued by most medical educators.		Historical, proposition of a curriculum / important	New insights about a course.
50	Francois Stainfort	2022	**Disciplinary Intersection Of Medicine And Business: A Novel Population Health Management Premedical Pathway For Medicine And Other Health Care Professions**	Population Health Management			No		N/a	Us	N/a	Students	N/a	Population health courses and business courses	New program	Population health management is a successful vehicle through which medical schools can work with business schools to create novel curricular programs that offer business of health knowledge to premedical students.		There are numerous factors motivating a curricular shift that integrates the disciplines of business and medicine; one of those is to improve the overall competency of students flowing into the valuable health care sector as well as into medical schools. Such structural and curricular innovations have the potential to tangibly improve the health care economy, and enhance the practice of medicine by training physicians who also understand the business of health.		When paired with the core basic sciences in a premedical curriculum, population health management students will have a diverse skillset to become effective agents of positive change.		Proposition of a new curriculum	
51	Carly Schnoebelen Et Al	2018	**Design And Evaluation Of A One-Semester General Chemistry Course For Undergraduate Life Science Majors**	Journal Of Chemical Education	Quantitative : comparison of exam scores, grades, and progression in organic chemistry between students who took the redesigned course vs. Traditional	Evaluate whether a biologically-focused one-semester general chemistry course (gcbf) better prepares life science students for organic chemistry than the traditional two-semester	Yes	4	Fall 2013-fall 2016	Us	Purdue university	Students	The majority of students who enroll in gcbf are freshmen in their first semester of undergraduate study. Enrollment has increased each year since the course was first offered in fall 2010, and in fall 2013 biology majors began taking the cours	Chemistry courses	Curriculum of chemistry			The gcbf course is a one-semester, five-credit general chemistry class designed specifically for life science majors. Less emphasis is placed on topics like electromagnetic radiation, quantum theory, crystal structures, nuclear chemistry, calorimetry, and colligative properties.	Premedical and life science students need a chemistry foundation that supports their understanding of biological systems, yet traditional general chemistry often overlooks key biologically relevant topics. In response, purdue university and the hhmi-sponsored nexus project developed the 1-2-1 curriculum, starting with a new one-semester general chemistry course (gcbf) tailored to bridge chemistry and biology.	Students benefit from the curriculum by being able to engage in undergraduate research, take upper-level courses, and take entrance exams such as the mcat earlier in their college careers as a result of completing biochemistry by the end of their sophomore year.	We are currently assessing student learning in biochemistry, particularly students' ability to apply their knowledge of chemistry to biological systems, which is an important goal of the curriculum.	Integrating competency-based preparation	
66	AAMC–HHMI Scientific Foundations for Future Physicians Committee (Co-chairs: Robert J. Alpern, Sharon Long; with ~25 national members)	2009	**Scientific Foundations for Future Physicians**	AAMC–HHMI Report	Not a study	To define core scientific competencies for premedical and medical education, shifting focus from rigid course requirements to competency-based	No		Committee convened 2007–2009; report published 2009	United States (national scope)	Association of American Medical Colleges; Howard Hughes Medical Institute; multiple universities represented (Yale, Stanford, Harvard, Baylor, etc.)	Premedical students and medical students (conceptual target populations, not direct study participants)	N/A (recommendations based on expert consensus, not student sample)	Premedical curriculum (biology, physics, chemistry, biochemistry, mathematics, social/behavioral sciences)	Curricular reform at national policy level (competency-based approach across multiple courses)		Encouraged replacement of rigid prerequisites with competency-based frameworks	Proposed competencies in biology, chemistry, physics, math/statistics, scientific reasoning, communication, ethics, lifelong learning	Shift from course-based prerequisites to competency outcomes; greater interdisciplinary integration; more flexibility in undergraduate curriculum	Calls from AAMC, HHMI, NRC, and reform literature about inadequacies in traditional premedical requirements. Established 8 competencies for medical graduates and 8 for entering students; influenced MCAT revision (2015) and national curricular changes	A blueprint for reform, not a prescriptive mandate; emphasizes flexibility, critical thinking, interdisciplinary approaches	Propose curricular reforms (competencies)	
69	Jason Adam Wasserman; Alisa A. Lopez; Roberta Knickerbocker; Antwione Haywood; Jonathan Kibble; Rebecca Fraser; Laura Fletcher	2025	**What Premedical Students Need to Succeed: Updated Premed Competencies for Entering Medical Students**	Academic Medicine (ahead of print)	Not a study	To update the AAMC's 2011 "Core Competencies for Entering Medical Students" in light of changes in health care, medical education, and society; to create an expanded competency framework for premedical	No		August 2022 – May 2023 (focus groups, drafting, revisions); published 2025	United States (national, multi-institutional scope)	AAMC; Oakland Univ. William Beaumont SOM; UCLA DGSOM; Univ. of Florida; Rosalind Franklin Univ.; Univ. of Central Florida; AAMC staff	Premedical students and entering medical students (target population); focus groups included prehealth advisors, faculty, admissions officers, and students	~23 focus groups; >100 medical schools represented; included advisors, admissions officers, medical students, faculty, and staff; broad national stakeholder input	Premedical education broadly (competency-based preparation rather than specific courses)	Large-scale policy-level change in defining entry competencies for medical school			Revised competency framework from 15 to 17 competencies; emphasized cultural humility, empathy, adaptability, and professional identity	Introduced expanded professional competencies: split "cultural competence" into cultural awareness + cultural humility; split "service orientation" into service orientation + empathy/compassion; emphasized growth mindset, resilience, teamwork & collaboration; integrated digital/virtual learning and DEI considerations	Changes in health care delivery, social determinants of health awareness, DEI imperatives, technological shifts (esp. post-COVID), need for holistic admissions frameworks	Established 17 updated "premed competencies" across 3 domains (professional, science, thinking/reasoning); more explicit articulation of personal and social attributes needed; will inform admissions, advising, and prehealth curricula	Propose curricular reforms (competencies)	Positive impact and improvement for future career development/ med school.
70	Brigitte Baldi; Jessica Utts	2015	**What Your Future Doctor Should Know About Statistics: Must-Include Topics for Introductory Undergraduate Biostatistics**	The American Statistician	Not a study	To highlight the increasing importance of statistical literacy in medicine and identify essential topics for an undergraduate biostatistics course aimed at premedical students	No		Literature review + curriculum proposal; published online Aug 2015	University of California, Irvine, USA	Dept. of Statistics, UC Irvine	Premedical undergraduates (conceptual target, not study participants)	N/A – no direct student sample; article synthesizes curriculum needs	Undergraduate introductory statistics/biostatistics	Curricular-level recommendation (design of specialized undergraduate course)	Proposed dedicated "Introductory Biostatistics for Premedical Students"		Suggested revising content of existing intro statistics to incorporate health-related examples and diagnostic testing	Drivers included MCAT 2015 changes emphasizing statistics/research methods; AAMC-HHMI 2009 competencies; evidence-based medicine movement	Physicians must learn to communicate risks and probabilities to patients; traditional intro stats courses insufficient; recommend health-centered examples	Defined "must-include" statistical concepts for future physicians (probability distributions, thresholds in disease definitions, diagnostic test PPV, conditional probability, context-based interpretation of significance, causality in research)	Propose curricular reforms	
52	Colleen Fried, Sandra Madar, And Carol Donley	2003	**The Biomedical Humanities Program: Merging Humanities And Science In A Premedical Curriculum At Hiram College**	Academic Medicine			No			Us	Hiram college	Students	Bimd majors	Lab and non lab	Curriculum: biomedical humanities (bimd) major	The bmd program aims to cultivate ethical, informed decision-making and cross-cultural communication among students while providing foundational science education in biology and chemistry for future medical studies, alongside			Abraham flexner's 1910 critique of medical education called for preparing physicians to handle "subtle elements" not addressed by science alone such as cultural sensitivity, ethical judgment, and strong interpersonal skills. At hiram college, this inspired the creation of a program for premedical students focused on	Faculty involved report personal growth in interdisciplinary teaching and reflection. The program attracts high-achieving students with varied career goals in medicine, law, education, and healthcare. The program is one of the most popular majors on campus despite its recent establishment.	Measuring its impact on students' humanity is difficult, but early indicators of success are strong	Not a study design of a new major biomedical humanities/ important	Positive feedback about the course.
53	David Muller	2014	**Flexmed: A Nontraditional Admissions Program At Icahn School Of Medicine At Mount Sinai**	American Medical Association Journal Of Ethics			No			Us	Icahn school of medicine at mount sinai	Students		Program	Talks about the humed program that was previously implemented at mount sinai	Humanities students were admitted without mcat or science prereqs and completed a science summer at mount sinai. The goal: promote a richer, less rigid education, reduce premed stress, and still achieve equal med school performance.			Current premed prep is outdated and poorly aligned with modern medicine's demands. It encourages competition over learning and doesn't equip students for real-world care, research, or teamwork. Reform should prioritize relevance, collaboration, and deeper thinking over rote memorization.	Humed students performed like their premed peers in most outcomes: clerkships, honors, leadership, and research. Slightly lower step 1 scores (6 points), but not clinically meaningful. Trend toward primary care/psychiatry noted. Flexmed enabled broader admissions criteria, valuing leadership, resilience, and non-academic		Not a study, proposition of a curriculum (humed)/ less important	Mixed outcomes
**Others (6)**																							
54	Ben Harder	2013	**Are Moocsthe Future Of Medical Education?**	British Medical Journal	Not a study.		No			Us	University of california-san francisco (ucsf) school of medicine.	Students and educators		"Massive open online courses" or moocs	Addition of MOOCs to the premedical curriculum. (not one specific course)	MOOCs (massive open online courses) could help fulfill premed competencies, especially in mathematics, by letting students learn at their own pace and revisit dense content.			Moocs are not meant to replace classroom learning but to offer more flexibility, especially for working adults and low-income students. Ultimately, they could widen access to medicine and help address the doctor shortage.	No relevant outcomes.	Moocs face major obstacles in medicine due to academic tradition, fear of new teaching methods, and financial concerns. There's limited data on their effectiveness, and faculty disagree on how medical topics should be taught. Despite this, their flexibility and convenience, especially for working professionals, make them a promising	Others/ not important: just a theoretical suggestion	No relevant outcomes
55	Kriya Gishen, Steven Ovadia, Samantha Arzillo,	2014	**The Current Format And Ongoing Advances Of Medical**	The Journal Of	Not a study.		No		N/a	Us	N/a	Residents/medical students		N/a	Not Applicable.	Not Applicable.			N/a	No relevant outcomes	N/a	N/a	No relevant outcomes
56	Samuel Kadavakollu, Boris Boyanovsky, Zea Moullet, Miho Yoshida, Mahboob Qureshi,	2021	**Undergraduate Pre-Requisite Coursework: Six Important Tips For Pre-Medical Students Considering Osteopathic**	International Journal Of Osteopathic Medicine	Not a study.		No		N/a	Us	N/a	Students		Both	Unclear about specific changes.	Only tips. No change took place. They only mentioned the UC Davis shorter anatomy sessions and their outcomes.			While aamc outlines core science and non-science prerequisites, successful medical school applicants must go beyond the basic list.	At UC Davis, undergraduate anatomy coursework, especially human gross anatomy and lab, significantly correlated with higher class rank and exam scores in the 172-hour medical anatomy		N/a	Mixed outcomes.
57	Charles Brenner	2012	**Changes In Chemistry And Biochemistry Education:Creative Responses To Medical College Admissions Test Revisions In The Age Of The Genome**	Biochemistry And Molecular Biology Education	Not a study.		No		2011	Us	Brandeis, iowa, dartmouth, pur due, texas, stanford, san jose state, haverford, macalester	Students, educators, administrators	N/a	Science courses (bio, chem, biochem)	Unclear about specific changes.	Proposals to replace the traditional two-year chemistry sequence with a one-year life-oriented course focused on biologically relevant molecular reactivity, introduce a mandatory biochemistry course, and require a lab course emphasizing research methods and statistics tailored to departmental strengths.			In response to the aamc's mr5 recommendations, the asbmb tasked leaders like suzanne pfeffer and dagmar ringe with developing focused premedical curriculum reforms in biology, chemistry, and biochemistry to better prepare future biomedical researchers and professionals.	No relevant outcomes.	As institutions implement life-oriented organic chemistry and asbmb-recommended curricula, it will be crucial to evaluate how these students perform in medical biochemistry relative to peers who followed the traditional chemistry sequence.	Revise biology, organic chemistry, biochemistry/ less important	No relevant outcomes
58	Robert M. Kaplan; Jason M. Satterfield; Raynard S. Kington	2012	**Building a Better Physician — The Case for the New MCAT**	New England Journal of Medicine	Not a study.	A 2004 Institute of Medicine report on "Improving Medical Education" and several years of evaluation identified a need to redesign the MCAT to better reflect physicians'	No		2009-2015	US	Association of American Medical Colleges (AAMC)	AAMC task force members, medical educators, admission officers; Premedical students; medical school admissions committees; undergraduate institutions	Not empirically sampled; authors discuss general trends and national policies affecting all U.S. premedical students	MCAT section	Justification of MCAT revision.	The new MCAT subtest entitled "Psychological, Social, and Biological Foundations of Behavior".			Behavioral and social factors profoundly affect health as both determinants and key factors in preventive or therapeutic interventions.	Aspiring doctors must understand patients' social and personal contexts, not just biology. Behavioral science must be clearly communicated and integrated into education and training. Medical schools need support to design evidence-based behavioral science curricula. Studying humanities is valuable but cannot replace rigorous training in behavioral and social sciences. These disciplines support both knowledge and essential skills needed in medical practice.	Medical schools will need guidance in developing and delivering curricula that meet high scientific standards while best serving the needs of patients and society.	MCAT revision, add a section/ others	New insight about an MCAT section.
61	Barr, Donald A.; Matsui, John	2008	**The 'Turning Point' for Minority Pre-Meds: The Effect of Early Undergraduate Experience in the Sciences on Aspirations to Enter Medical School of Minority Students at UC Berkeley and Stanford University. Research & Occasional Paper Series: CSHE.20.08**	Center for Studies in Higher Education	Mixed: Surveys were conducted at the start of freshman year and again at the end of sophomore year at both universities. One-on-one interviews were also conducted with 63 students between sophomore and senior	See which course makes premeds lose interest.	Yes	5	2002-2005	US	UC Berkeley and Stanford	undergraduate students	423 Berkeley freshmen students and 362 Stanford freshmen students		Unclear about specific changes.	The article does not explicitly state how the course or curriculum should be changed, but it emphasizes that chemistry courses need reform, as they are currently filtering out students who might otherwise become excellent physicians.			It appears that, over the four years of the undergraduate experience, Stanford loses fifty or more URM students per class who once had hoped to become a physician. It is not that these students fail to graduate; rather, they abandon premedical studies and pursue other career trajectories.	97% of URM students and 65% of non-URM students cited at least one course that discouraged them. Chemistry courses, especially organic and inorganic, were the most commonly cited discouraging factor. Chemistry was named the single most discouraging course by 71% of URM students and 55% of non-URM students. The findings suggest early science coursework, particularly chemistry, plays a major role in premed attrition.	It falls disproportionately on students from underrepresented racial and ethnic groups, many of whomenter the university from a position of educational disadvantage.	Not important	Not relevant to our common outcomes.
