# Supplementary material for: Retuning the Premedical Compass in American Programs Worldwide: Scoping Review
Source: JMIR Med Educ. 2026 Apr 9;12:e85002. doi: 10.2196/85002 (PMC13064960; doi:10.2196/85002)
Supplement: Multimedia Appendix 6 [file mededu-v12-e85002-s006.pdf]

					SCREENING QUESTIONS		1. QUALITATIVE STUDIES					4. QUANTITATIVE DESCRIPTIVE STUDIES					5. MIXED METHODS STUDIES				
ID	Authors	Year	Title	Overall Appraisal	S1. Are there clear research questions?	S2. Do the collected data allow to address the research questions?	1.1. Is the qualitative approach appropriate to answer the research question?	1.2. Are the qualitative data collection methods adequate to address the research question?	1.3. Are the findings adequately derived from the data?	1.4. Is the interpretation of results sufficiently substantiated by data?	1.5. Is there coherence between qualitative data sources, collection, analysis and interpretation?	4.1. Is the sampling strategy relevant to address the research question?	4.2. Is the sample representative of the target population?	4.3. Are the measurements appropriate?	4.4. Is the risk of nonresponse bias low?	4.5. Is the statistical analysis appropriate to answer the research question?	5.1. Is there an adequate rationale for using a mixed methods design to address the research question?	5.2. Are the different components of the study effectively integrated to answer the research question?	5.3. Are the outputs of the integration of qualitative and quantitative components adequately interpreted?	5.4. Are divergences and inconsistencies between quantitative and qualitative results adequately addressed?	5.5. Do the different components of the study adhere to the quality criteria of each tradition of the methods involved?
3	Rachel Conrad Bracken, Ajay Major, Aleena Paul, Kirsten Ostherr	2021	**Reflective Writing About Near-Peer Blogs: A Novel Method For Introducing The Medical Humanities In Premedical Education**	Include	Yes	Yes	Yes	Yes	Yes	Yes	Yes										
5	Gia Merlo	2021	**Mpro: A Professionalism Curriculum To Enhance The Professional Identity Formation Of University Premedical Students**	Include	Yes	Yes	Yes	Can't tell	Yes	Yes	Can't tell										
7	Jason Wilson	2019	**Patient Shadowing: A Useful Research Method, Teaching Tool, And Approach To Student Professional Development For Premedical Undergraduates**	Include	Yes	Yes	Can't tell	Can't tell	Yes	Can't tell	Can't tell										
10	Malvitz, Madelyn; Khan, Noreen; Morgenstern, Lewis B	2024	**College Preparation For A Medical Career In The United States.**	Include	Yes	Yes	Yes	Can't tell	Yes	Yes	Can't tell										
12	Milos Savic,Paxtonmartin;	2018	**The Perceived Versus Actual Use Of Mathematics In Medicine According To Pre-Medicine Students And Practicing Physician**	Include	Yes	Yes	Yes	Yes	Yes	Yes	Can't tell										
13	Natalie K. Cooke, Sarah L. Ash, L. Suzanne Goodell And Kelsey L. Wilson	2015	**Qualitative Assessment Of Pre-Healthcare Undergraduates' Perceptions Of Childhood Obesity To Inform Premedical Curricular Changes**	Include	Yes	Yes	Yes	Yes	Yes	Can't tell	Yes										
22	Rebecca Thomas, Taylor Yancey, Chad Skidmore, Neal Ferrin, Isain Zapata, Jennifer Williams, Nena Lundgreen Mason	2021	**The Effects Of Pre-Medical Anatomy And Clinical Experiences On Medical School Anatomy-Related Academic Performance**	Include	Yes	Yes	Yes	Yes	Yes	Can't tell	Can't tell										
37	Jon-Philippe K Hyatt & Stephen A Hurst	2010	**Novel Undergraduate Physiology Laboratory Using A Human Patient Simulator**	Include	Yes	Yes	Can't tell	Can't tell	Yes	Yes	Yes										
62	Jessica Watkins; Janet E. Coffey; Edward F. Redish; Todd J. Cooke	2012	**Disciplinary authenticity: Enriching the reforms of introductory physics courses for life-science students**	Include	Yes	Yes	Yes	Yes	Yes	Yes	Yes										
9	Lauren D. Olsen	2016	**"It'S On The Mcat For A Reason": Premedical Students And The Perceived Utility Of Sociology**	Include	Yes	Yes	Yes	Yes	Yes	Yes	Yes										
67	Afghani B.; Manalang J.; Salazar A.; Lee J.; Kim K.	2013	**Should cadaver lab be introduced to premedical students in an effort to promote their interest towards health careers?**	Include	Yes	Yes						Yes	Can't tell	Yes	Can't tell	Yes					
63	Christian Moro; Michelle McLean; Charlotte Phelps	2025	**Doing justice when incorporating sustainability into pre-medical curricula**	Include	Yes	Yes						Yes	Can't tell	Yes	Can't tell	Yes					
68	Kavitha T. Ram; Howard A. Blanchette	2015	**How Should We Teach Physicians the Language of Statistics?**	Include	Yes	Yes						Yes	Can't tell	Yes	Yes	Yes					
2	Courtney M. Smalley, Vaughn Browne, Bonnie Kaplan, Brian Russ, Juliana Wilson, Resa E. Lewiss	2016	**A Course For Pre–Medical Professions Students Using Point-Of-Care Ultrasound**	Include	Yes	Yes						Yes	Yes	Yes	Yes	Yes					
4	Joanne Tran,1 Leo Meller,1 Vy Le,1 Jasmine Tam,1 Andrea Nicholas.	2024	**Behavioral Assessment Of Soft Skill Development In A Highly Structured Pre-Health Biology Course For Undergraduates**	Include	Yes	Yes						Yes	Can't tell	Yes	Yes	Yes					
8	Joseph P. Forester, David L. Mcwhorter,* And Maria S. Cole:	2002	**The Relationship Between Premedical Coursework In Gross Anatomy And Histology And Medical School Performance In Gross Anatomy And Histology**	Include	Yes	Yes						Yes	Yes	Yes	Can't tell	Yes					
59	Stevens, Courtney; Witkow, Melissa R	2014	**Training scientific thinking skills: Evidence from an MCAT2015-aligned classroom module**	Exclude	Yes	Yes						Yes	Can't tell	No	Can't tell	No					
20	Marlene D. Louro, Grace Meegan, Lauren R. Rudin, Nathan E. Thompson	2023	**Students With Prior Anatomy Experience Start Out Stronger In Medical School Gross Anatomy**	Include	Yes	Yes						Yes	Yes	Yes	Yes	Yes					
25	David Muller, And Nathan Kase	2010	**Challenging Traditional Premedical Requirements As Predictors Of Success In Medical School: The Mount Sinai School Of Medicine Humanities And Medicine Program**	Include	Yes	Yes						Yes	No	Yes	Can't tell	Yes					
27	William W. Kennerly, Kimberley A. Frederick, And Kelly Sheppard	2020	**General Chemistry In Just One Semester For All Majors**	Include	Yes	Yes						Yes	Yes	Yes	Can't tell	Yes					
34	Cathryn A. Peterson, Richard P. Tucker	2005	**Undergraduate Coursework In Anatomy As A Predictor Of Performance: Comparison Between Students Taking A Medical Gross Anatomy Course Of Average Length And A Course Shortened By Curriculum Reform**	Include	Yes	Yes						Yes	Can't tell	Yes	Yes	Yes					
35	Kathryn C. Bolduc, Shannonl. Mccollough , Angelak.Stoeckman	2022	**From Classroom To Clinic: Biochemistry Lab For Pre-Health Majors**	Include	Yes	Yes						Yes	No	Yes	Can't tell	Yes					
36	David M. Harris, Christine Bellew, Zixi J. Cheng, Juan C. Cendán.And Jonathan D. Kibble	2014	**High-Fidelity Patient Simulators To Expose Undergraduate Students To The Clinical Relevance Of Physiology Concepts**	Include	Yes	Yes						Yes	No	Yes	Can't tell	Yes					
43	Linnea Dixson, Briana Pomales, Mehrtash Hashemzadeh, And Mehnroosh Hashemzadeh	2022	**Is Organic Chemistry Helpful For Basic Understanding Of Disease And Medical Education?**	Include	Yes	Yes						Yes	Can't tell	Yes	Can't tell	Yes					
51	Carly Schnoebelen Et Al	2018	**Design And Evaluation Of A One-Semester General Chemistry Course For Undergraduate Life Science Majors**	Include	Yes	Yes						Yes	Yes	Yes	Yes	Yes					
60	Claudia Da Silva Carvalho	2024	**Knowledge and Perception of Antibiotic Resistance and Stewardship among Pre-Health and Agriculture Undergraduate Students**	Include	Yes	Yes											Yes	Yes	No	Yes	Can't tell
6	Hursuong Vongsachang, Sachi Oshima, Christine Nguyen, Suzanne Gaulocher, N. Ewen Wang;	2020	**Undergraduate Premedical Student Perceptions Of An Emergency Department–Based Social Needs Screening Program**	Include	Yes	Yes											Yes	Yes	Yes	Yes	Yes
14	Peter Kondrashov	2017	**Premedical Anatomy Experience And Student Performance In Medical Gross Anatomy**	Include	Yes	Yes											Yes	Yes	Can't tell	Yes	Yes
31	Pablo Rodríguez Del Pozo, And Joseph J. Fins	2005	**The Globalization Of Education In Medical Ethics And Humanities: Evolving Pedagogy At Weill Cornell Medical College In Qatar**	Include	Yes	Yes											Yes	Yes	Yes	Yes	Can't tell
33	Tyler J Stodola And Sean P Bruna	2021	**Medical Anthropology Courses And Concepts Tested On The Mcat: A Content Analysis Of 40 U.S. Course Syllabi**	Include	Yes	Yes											Can't tell	Can't tell	Yes	Yes	Yes
38	Mochrie, S.G.J.	2016	**Vision And Change In Introductory Physics For The Life Sciences**	Include	Yes	Yes											Yes	Yes	Yes	Yes	Yes
39	Sherri C. Young	2016	**Comparable Educational Benefits In Half The Time: An Alternating Organic Chemistry Laboratory Sequence Targeting Prehealth Students**	Include	Yes	Yes											Yes	Can't tell	Yes	Can't tell	Can't tell
64	Michael C. Lotspeich-Yadao; Kimberly Trevino-Boissel; Cara Hoekstra	2025	**Integrating Experiential Learning in Introduction to Sociology: Cultivating the Sociological Imagination in Premedicine Students**	Include	Yes	Yes											Yes	Yes	Can't tell	Yes	Yes
41	E. F. Redish	2014	**Nexus/Physics: An Interdisciplinary Repurposing Of Physics For Biologists**	Include	Yes	Yes											Yes	Can't tell	Yes	Yes	Yes
44	Gerd Kortemeyer	2007	**The Challenge Of Teaching Introductory Physics To Premedical Students**	Include	Yes	Yes											Yes	Yes	Yes	Yes	Can't tell
45	Ryan L. Stowe And Melanie M. Cooper	2017	**Practicing What We Preach: Assessing "Critical Thinking" In Organic Chemistry**	Exclude	Yes	Yes											Yes	Yes	Can't tell	No	No
46	Elliot Mylott, Ellynne Kutschera, Justin C. Dunlap, Warren Christensen, Ralf Widenhorn	2016	**Using Biomedically Relevant Multimedia Content In An Introductory Physics Course For Life Science And Pre-Health Students**	Include	Yes	Yes											Yes	Yes	Yes	Can't tell	Can't tell
61	Barr, Donald A.; Matsui, John	2008	**The 'Turning Point' for Minority Pre-Meds: The Effect of Early Undergraduate Experience in the Sciences on Aspirations to Enter Medical School of Minority Students at UC Berkeley and Stanford University. Research & Occasional Paper Series: CSHE.20.08**	Exclude	Yes	Yes											Yes	Can't tell	Yes	No	Can't tell
1	Kim Rheinlander, Dorothy Wallace	2011	**Calculus, Biology And Medicine: A Case Study In Quantitative Literacy For Science Students**	Include	Yes	Yes											Can't tell	Yes	Yes	Can't tell	Yes
